# Preparation and Characterization of an Engineered FGF1 Conjugated to ^161^Tb for Targeting of FGFRs

**DOI:** 10.1021/acsomega.4c09179

**Published:** 2025-02-06

**Authors:** Linlin Song, Michal Kostas, Jon K. Laerdahl, Marie Skálová, Tereza Janská, Asta Juzeniene, Svein Ræstad, Alexander Krivokapic, Georgios N. Kalantzopoulos, Jaroslav Soltes, Martin Vlk, Jan Kozempel, Sindre Hassfjell, Jørgen Wesche

**Affiliations:** † Department of Tumor Biology, Institute for Cancer Research, The Norwegian Radium Hospital, Oslo University Hospital, Montebello, Oslo 0379, Norway; ‡ Centre for Cancer Cell Reprogramming, Institute of Clinical Medicine, Faculty of Medicine, University of Oslo, Montebello, Oslo 0379, Norway; § Department of Microbiology, Oslo University Hospital, Rikshospitalet, Oslo 0424, Norway; ∥ ELIXIR Norway, Department of Informatics, University of Oslo, Oslo 0316, Norway; ⊥ Faculty of Nuclear Sciences and Physical Engineering, Czech Technical University in Prague, Břehová 7, Prague 1 110 00, Czech Republic; # Department of Radiation Biology, Institute for Cancer Research, The Norwegian Radium Hospital, Montebello, Oslo, 0379 Norway; ¶ Department of Tracer Technology, Institute of Energy Technology, Instituttveien 18, Kjeller 2007, Norway; ∇ Centrum výzkumu Řež s.r.o., Hlavní 130, Řež, Husinec 250 68, Czech Republic; ○ Thor Medical, Karenslyst allé 9C, Oslo 0278, Norway; ⧫ Department of Molecular Medicine, Institute of Basic Medical Sciences, University of Oslo, Oslo 0372, Norway

## Abstract

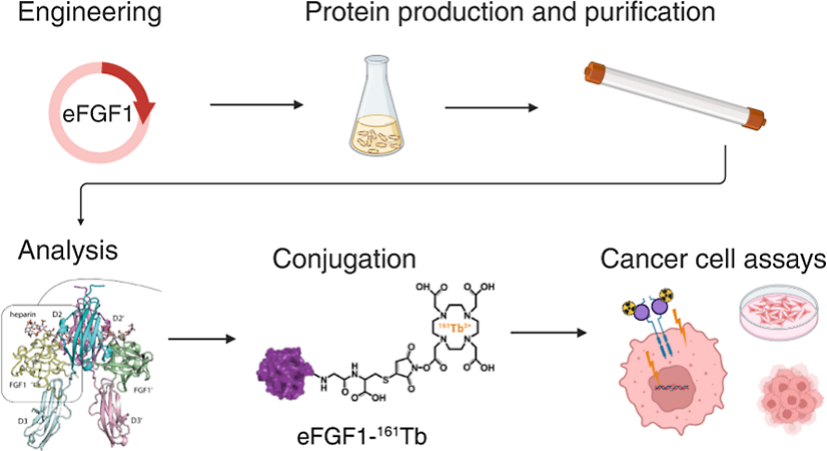

The fibroblast growth factor receptor family members, FGFR1-4, are frequently overexpressed in various solid tumors, including breast cancer and sarcomas. This overexpression highlights the potential of the family of FGFRs as promising targets for cancer therapy. However, conventional FGFR kinase inhibitors often encounter challenges such as limited efficacy or drug resistance. In this study, we pursue an alternative strategy by designing a conjugate of the FGFR ligand FGF1 with the radioisotope ^161^Tb, for targeted therapy in FGFR-overexpressing cancer cells. FGF1 was engineered (eFGF1) to incorporate a single cysteine at the C terminus for site-specific labeling with a DOTA chelator. eFGF1-DOTA was mixed with the radioisotope ^161^Tb under mild conditions, resulting in a labeling efficiency above 90%. The nonradioactive ligands were characterized by mass spectrometry, while radioligands were characterized by thin-layer chromatography. The targeting function of the radioligands was assessed through confocal microscopy, flow cytometry, and Western blot analysis, focusing on binding to cancer cells and the activation of downstream signaling pathways related to FGFR. When compared to MCF-7 and RD cell lines with low FGFR expression, eFGF1-DOTA-Tb[^161^Tb] radioligands demonstrated significantly higher accumulation in FGFR-overexpressing cell lines (MCF-7 FGFR1 and RMS559), leading to enhanced cytotoxicity. Besides radionuclides, eFGF1 can also deliver doxorubicin (DOX) into cancer cells. Considering these characteristics, eFGF1-DOTA-Tb[^161^Tb] and eFGF1-DOX emerge as promising candidates for FGFR-targeted cancer therapy, and further evaluation in vivo is warranted.

## Introduction

The fibroblast growth factor receptor (FGFR) family consists of four transmembrane receptors, FGFR1-4, which play essential roles in development, metabolism, tissue homeostasis, and disease pathogenesis.^1^ Fibroblast growth factors (FGFs) are polypeptide growth factors that bind to and activate FGFRs. The FGF signaling system is highly relevant for the development, progression, and metastasis of many types of cancer.^2^ According to previous analyses, the dysregulation of FGFRs has been detected in 5–10% of human cancers,^3^ including 7–23% FGFR1/2 overexpression in breast cancer,^4^ 20% FGFR1 overexpression in nonsmall cell lung cancer,^5^ and 7–8% FGFR4 overexpression in rhabdomysarcoma.^6^ This indicates that the FGFR family is a promising receptor for targeting in cancer therapy. The majority of anti-FGFR targeting cancer therapies rely on small molecule inhibitors. For example, erdafitinib^7,8^ has already been approved for treatment of FGFR3-altered urothelial cancer, and pemigatinib^8,9^ has been approved for treatment of FGFR2-fusion driven cholangiocarcinoma and myeloid/lymphoid neoplasms. However, secondary kinase mutations causing resistance to tyrosine kinase inhibitors (TKIs) frequently occur in urothelial cancers and cholangiocarcinoma.^10,11^ Also, TKIs have been shown to have limited efficacy in breast cancer despite FGFR1 overexpression,^12^ probably because these cancers are not sufficiently oncogene-addicted to the FGFR1 signal.

Therefore, some studies have attempted to target the extracellular part of FGFRs through antibodies^13,14^ or modified ligands.^15^ In contrast to TKIs, where the cancer cells need to be oncogene-addicted to the target kinase, overexpressed receptors can be targeted as long as they are surface exposed. Kinase inhibitor resistance mutations in the intracellular part will not have an impact. FGF ligands or FGFR-directed antibodies have been conjugated to highly toxic drugs to specifically kill cancer cells overexpressing FGFRs.^14,15^ Among the ligands of the FGF family, FGF1 can bind to all four types of FGFRs, as well as heparan sulfate proteoglycans (HPSGs) and heparin,^12,16,17^ which indicates that FGF1 could be a potential vector for targeted drug delivery for several cancer types.

Certain therapeutic radionuclides show high potential in medical applications, including the α-emitters ^225^Ac, ^212^Pb, ^223^Ra or β-emitters ^177^Lu, ^161^Tb, ^89^Sr because they emit particles with high energy, which have been found suitable for both tumor diagnosis and tumor therapy.^17−19^ After conjugation with targeting vectors like antibodies, nanobodies, or peptides, the radionuclides could work as a magic bullet binding to markers on the tumor cells and then destroy them.^20−22^ This strategy has been called targeted radionuclide therapy. ^161^Tb is a radionuclide with a half-life of 6.9 days and emits low-energy β^–^particles ( = 154 keV) with a maximal tissue range of 0.29 mm, which is similar to the clinically applied radionuclide ^177^Lu (half-life of 6.7 days,  = 134 keV). Besides that, ^161^Tb shows better therapeutic potential by emitting a large amount of Auger/conversion electrons with an energy ≤50 keV (∼12.4 e^–^, 46.5 keV per decay), which could potentially cause damage to subcellular structures in tumor cells.^23−25^

**Figure 1:**
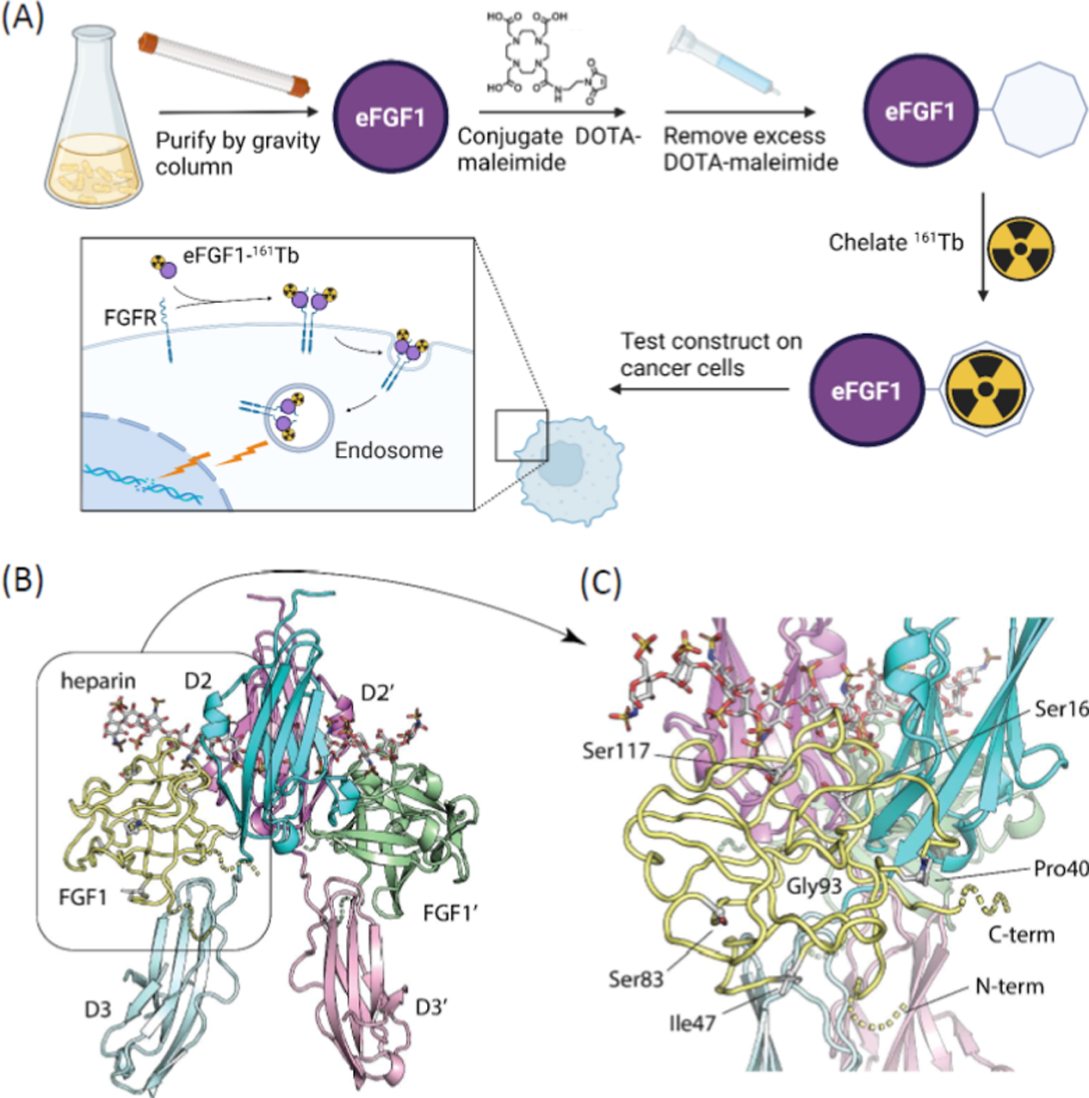
(A) Schematic representation of the design and production process of eFGF1-^161^Tb conjugates for targeted cancer cell therapy. Created in https://BioRender.com. (B) Structure model of the FGF1: FGFR1: heparin complex dimer. The FGFR1 Ig-like domains 2 (D2) and 3 (D3) interacting with FGF1 (yellow) are shown in dark and light cyan, respectively. In the second complex of the dimer, with approximate *C*_2_ symmetry, FGF1 (green) is interacting with FGFR1 D2′ (dark pink) and D3′ (light pink). Heparin is shown in stick rendering. The structurally disordered N- and C-termini of the FGF1 proteins, shown in dashed rendering, do not appear to be interacting with either FGFR1 or heparin. (C) FGF1 C-termini with the radiolabel DOTA-^161^Tb is located far from all other macromolecules in the complex dimer (>10 Å). All mutated residues in the engineered C16S Q40P S47I C83S H93G C117S FGF1 are highlighted.

In this study, we engineered the FGF1 ligand and radiolabeled it with ^161^Tb for targeting FGFR-overexpressing cancer cells. The eFGF1 vector was engineered to allow site-specific conjugation of maleimide-DOTA and chelation of the ^161^Tb radionuclide. Site-specific labeling was chosen to avoid conflict of the conjugation to binding of the ligand to its receptor. Site-specific labeling could also be advantageous to avoid batch–batch variation in the preparation of the final conjugate. As the low stability of wild-type FGF1 limited its application as a targeting vector, we introduced three point mutations (Q40P/S47I/H93G), which were reported to extend the in vivo half-life of FGF1 from 0.26 to 150 h.^26^ To test the targeting effect of the conjugates, we chose a breast cancer cell line and a rhabdomyosarcoma cell line because these cancers often overexpress FGFRs. The schematic in Figure 1A indicates that eFGF1-DOTA-Tb [^161^Tb] (eFGF1-^161^Tb in abbreviation) can bind to FGFR receptors, endocytose into cells by receptor-mediated internalization, be transported into the inner parts of cells, and irradiate the DNA double helix to kill tumor cells. Compared to the control cell line, eFGF1-^161^Tb showed much higher uptake and significantly enhanced cytotoxicity on both FGFR1 and FGFR4 overexpressing cell lines, which suggests that eFGF1-^161^Tb is a promising candidate for targeted cancer therapy.

## Results

### Construction and Expression of an Engineered FGF1-Based Vector

To make a vector suitable for targeting cancer cells overexpressing FGFRs, we generated an engineered version of the FGF1 protein, a natural ligand for all four human FGFRs (FGFR1-4). First, to ensure high thermal stability of the FGF1-based vector, we introduced three previously identified point mutations that dramatically enhance protein stability (Q40P/S47I/H93G) in a truncated variant of full-length FGF1.^26^ The N-terminal truncation and three mutations have previously been shown to not negatively affect cell–surface FGFR binding or mitogenic activity.^26,27^ Then, we added a Gly–Cys dipeptide at the FGF1 C-terminus to allow for labeling with maleimide-based linkers (Figure S1). A structural model of the FGF1: FGFR1: heparin complex indicates that labeling of FGF1 at the C-terminus is unlikely to interfere with the interaction between FGF1 and FGFRs or heparin (Figure 1B,C). We also mutated the three naturally occurring Cys residues in FGF1 (C16S, C83S, and C117S) to allow for specific and site-directed labeling at the FGF1 C-terminus. Mutation of these Cys residues has previously been shown to not affect the binding ability of FGF1 to its receptors.^28^ We ordered synthetic DNA harboring these changes and cloned the DNA fragment coding for the construct denoted eFGF1, into an expression vector (Figure S2). The construct was expressed in *E. coli,* and the protein was easily purified by a heparin-Sepharose gravity column (Figure S3). We routinely obtained 10 to 20 mg of eFGF1 from 1 L of cultured bacteria, and this eFGF1 ligand shows serum stability longer than 10 days (Figure S4). Fluorescence spectroscopy analyses of eFGF1 indicated that the tertiary structure was intact, suggesting correct folding of the mutant and a native structure (Figure S5). To test for signaling, we incubated eFGF1 with MCF-7 cells overexpressing FGFR1 (MCF-7 FGFR1) and used Western blotting and phospho-specific antibodies to monitor activated receptors (pFGFR) and downstream signaling (pPLCγ and pERK). Signaling of the engineered version was very similar to that of wild-type FGF1, indicating that binding of eFGF1 to FGFRs was not much affected (Figure S6).

### Intracellular Uptake and Infiltration in Spheroids of Engineered eFGF1

To study binding and selective internalization of the engineered eFGF1 in FGFR overexpressing cells, we used four cell lines: MCF-7, MCF-7 FGFR1, RD, and RMS559. The MCF-7 breast cancer cell line expresses very low levels of FGFR1, and we therefore also used a previously generated MCF-7 cell line stably overexpressing FGFR1^15^ (MCF7-FGFR1, Figure S8). The rhabdomyosarcoma cell line RMS559 expresses high amounts of FGFR1 and FGFR4. The RD cell line expresses very low levels of FGFRs and served as a negative control (Figures S8 and S9).

**Figure 2:**
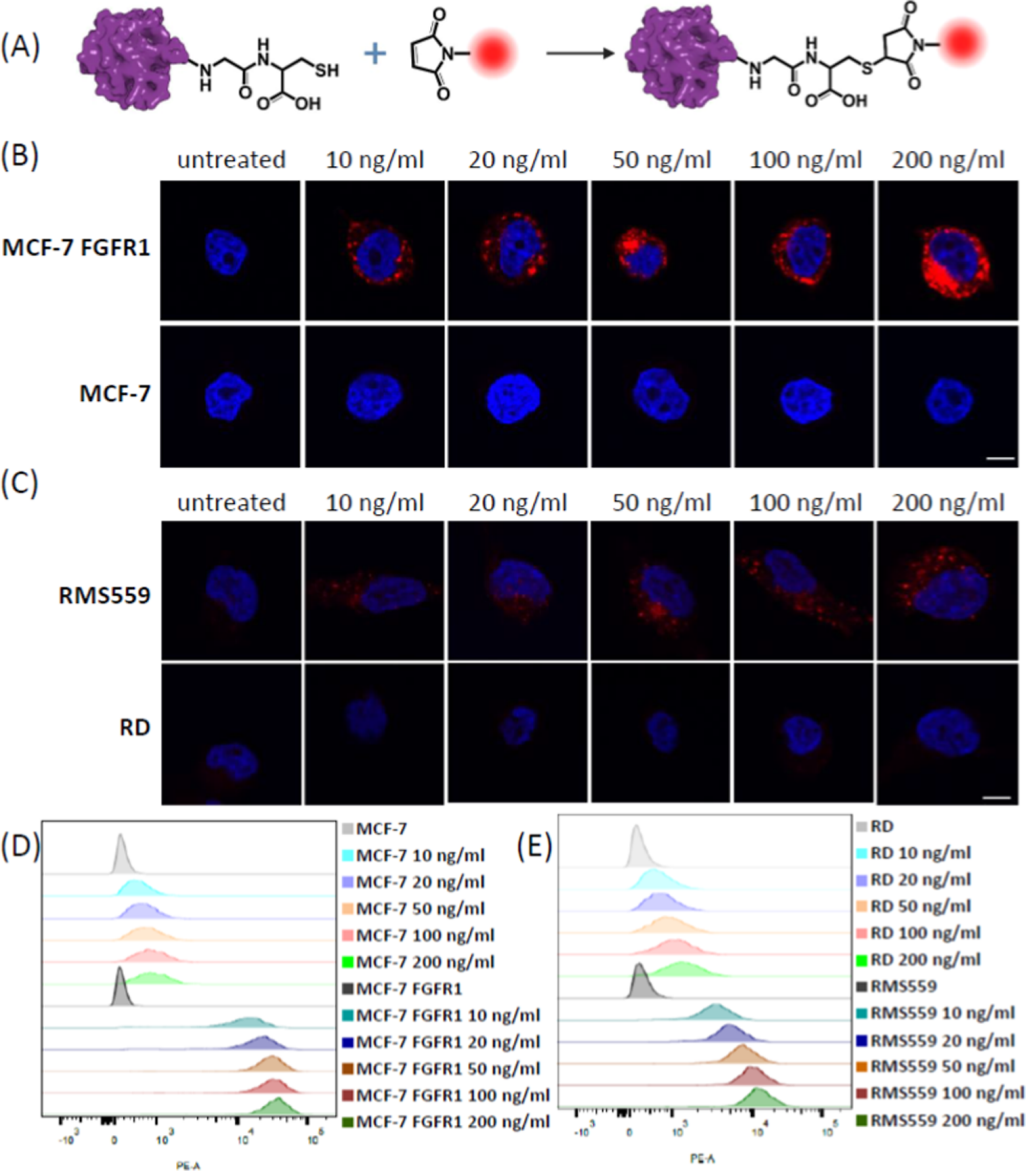
Evaluation of eFGF1 ligand internalization in FGFR overexpressed cells. (A) Chemical structure of the site-specific labeling strategy of eFGF1-DL550 conjugates. FGF1 was engineered with one cysteine on the C-terminus, which could react with the maleimide group. (B,C) Representative confocal images of eFGF1-DL500 internalization in MCF-7 FGFR1/MCF-7 cells and RMS559/RD cells after 2 h incubation (excitation wavelength: 568 nm for DL550 labeled eFGF1, pseudocolor red; 405 nm for nucleus, pseudocolor blue; scale bars, 10 μm). (D,E) Flow cytometry analysis of MCF-7 FGFR1/MCF-7 cells and RMS559/RD cells after 2 h incubation with eFGF1-DL550.

We labeled eFGF1 with maleimide DyLight 550 (DL550) to visualize the protein by microscopy (Figure 2A) and confirmed the labeling by gel electrophoresis (Figure S7). Cells were incubated with different concentrations of eFGF1-DL550 at 37 °C for 2 h and immobilized on glass slides, followed by confocal microscopy. We observed high uptake of eFGF-DL550 in MCF-7 FGFR1 cells (Figure 2B), while the signal in MCF-7 cells was hardly detected (Figure 2B). Flow cytometry analysis further demonstrated that eFGF1 was only able to internalize into MCF-7 FGFR1, but not MCF-7 (Figure 2D). We then analyzed the uptake of eFGF1 in rhabdomyosarcoma cells with the same method and observed that the internalization of eFGF1 in RMS559 was considerably higher than that in RD cells (Figure 2C,E), which was consistent with the FGFR expression levels in both cell lines (Figures S8 and S9). Taken together, the internalization of eFGF1 was mediated specifically by FGFRs.

**Figure 3:**
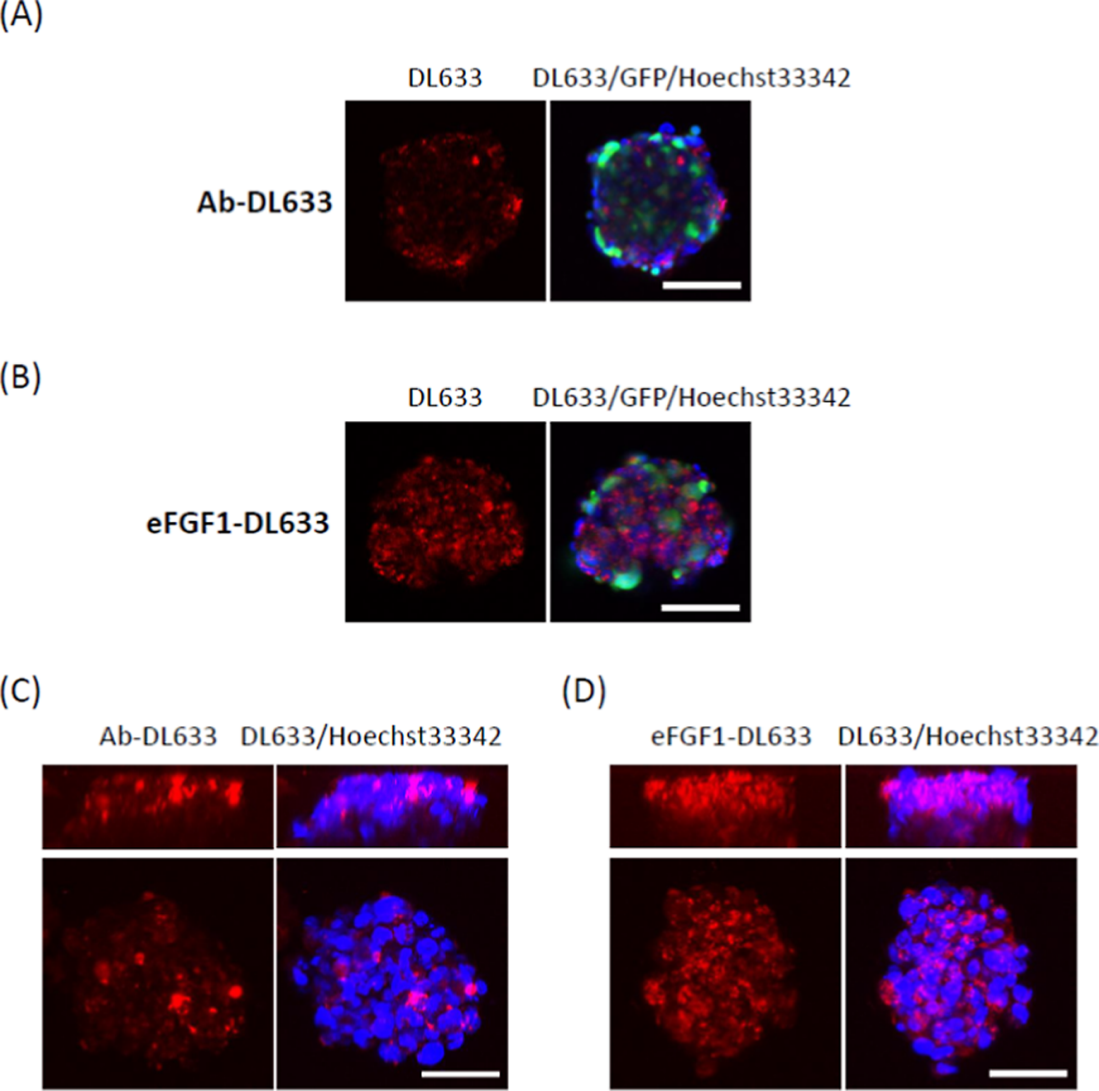
Comparison of the eFGF1 ligand and FGFR1 antibody infiltration in 3D spheroids. (A,B) Confocal fluorescence imaging of GFP-RMS559 spheroids after treatment with 10 μg/mL FGFR1 antibody-DL633 and 2 μg/mL eFGF1-DL633 for 24 h. (C,D) Representative top and orthogonal 3D-volume projections of the spheroids from Z-stack confocal imaging after treatment with FGFR antibody-DL633 and eFGF1-DL633 for 24 h. (Excitation wavelength: 633 nm for DL633 labeled eFGF1 and Ab, pseudocolor red; 488 nm for GFP, pseudocolor green; 405 nm for nucleus, pseudocolor blue, scale bars, 100 μm).

To investigate the internalization of eFGF1 in a 3D tumor model, we generated RMS559 stably expressing GFP protein (GFP-RMS559) and cultured them into spheroids by seeding cells in ultralow attachment plates and culturing for 3 days,^29^ until the spheroids reached a size of 250–300 μm. eFGF1 and a monoclonal FGFR1 antibody targeting the extracellular part of the receptor were labeled with NHS DyLight 633 (DL633) and incubated with the spheroids for 24 h. The spheroids were imaged using a Nikon spinning disc confocal microscope with equal fluorescence settings (same laser power and gain value). As shown in Figure 3A,C, the FGFR1 antibody mainly binds and internalizes into the cells on the surface of spheroids, while the signal in the spheroid center is much weaker. The fluorescence of eFGF1-DL633 is homogeneous in the cross-section of the spheroids, which indicates that eFGF1 is able to infiltrate deep into the 3D tumor model (Figure 3B,D), probably because of its smaller size (Figure S4). Visualized by the 3D Z-stack mode, we could see that the signal of eFGF1-DL633 in the spheroids is much higher than that of FGFR Ab-DL633 from both top and orthogonal projections (Figure 3C,D). The data indicate that eFGF1 is able to bind strongly to FGFR receptors and infiltrate 3D microtumors.

### Synthesis and Characterization of eFGF1-^161^Tb Radioligands

**Figure 4:**
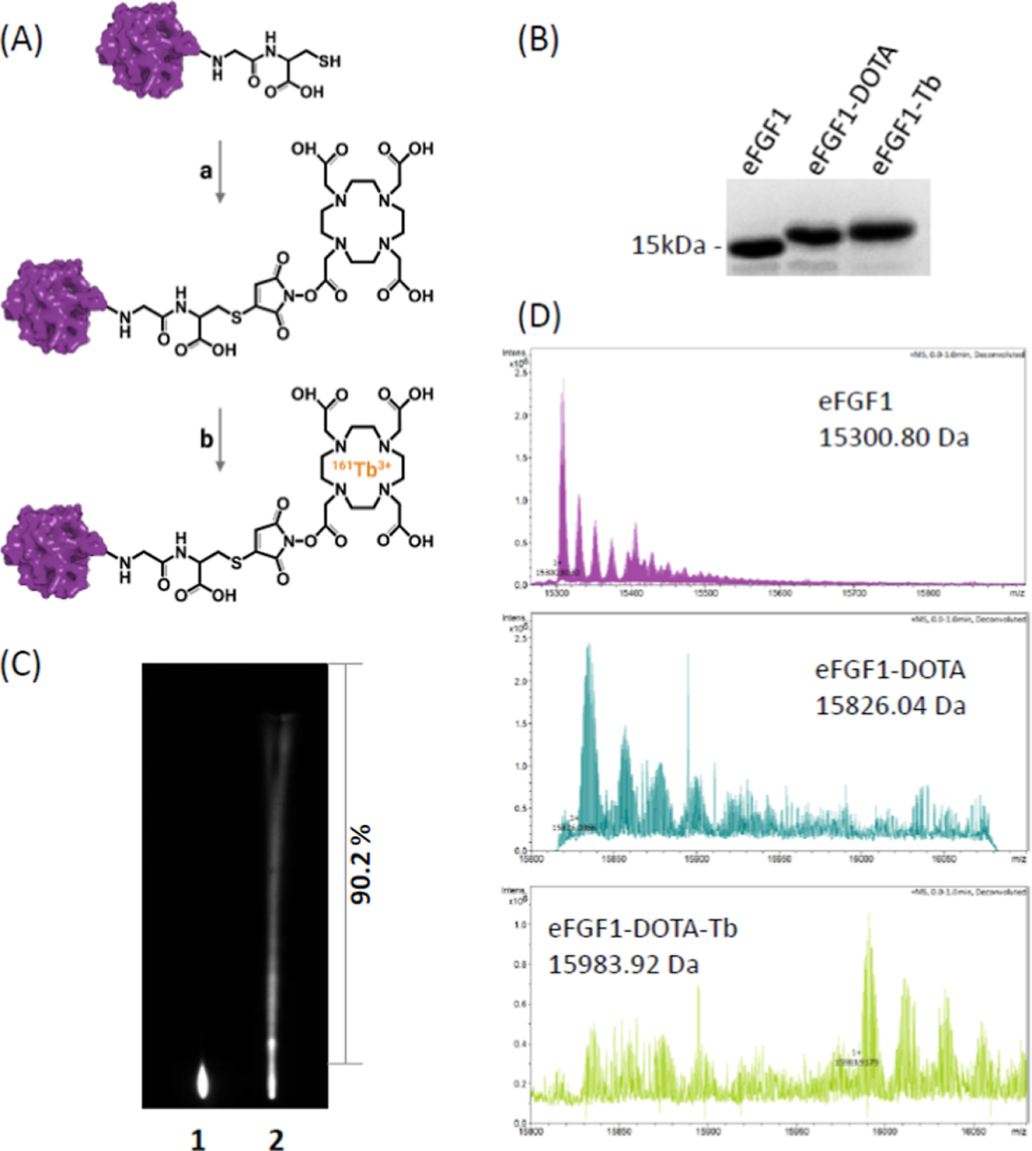
Characterization of eFGF1-Tb conjugates. (A) Molecular structure design and conjugation strategy of eFGF1-Tb conjugates. (a) DOTA-maleimide, 0.5 M NaOAc pH7.4, 23 °C, 1 h; (b) TbCl_3_, 0.5 M NaOAc pH6.0, 37 °C, 16 h. (B) PAGE gel electrophoresis analysis of eFGF1, eFGF1-DOTA, and eFGF1-Tb. (C) Representative images of the thin-layer chromatography test of eFGF1-^161^Tb conjugates. Line 1 indicates free ^161^Tb^3+^, and Line 2 indicates the production of eFGF1-^161^Tb conjugates. (D) Comparison of the molecular weight between eFGF1, eFGF1-DOTA, and eFGF1-Tb by MS.

To make eFGF1-^161^Tb, we first linked maleimide-DOTA to eFGF1 by a Michael-type addition reaction between the maleimide and free thiol on the C-terminal end of eFGF1 (Figure 4A). We first optimized the eFGF1-Tb production with a nonradioactive Tb isotope (cold Tb). eFGF1-Tb was generated by incubating eFGF1-DOTA with Tb^3+^ in pH 6.0 buffer at 37 °C for 16 h. The reaction was performed at mildly changed pH, because FGF1 unfolds at low pH. The formation of eFGF1-Tb was confirmed by gel electrophoresis and LC–MS (Figure 4B,D). The molecular weight of eFGF1 is 15300.8 Da, which is consistent with our designed amino acid residue sequence. The final product eFGF1-Tb molecular weight increased by 683.1 Da, which is exactly the sum of one maleimide-DOTA molecule and one Tb cation, indicating that a single maleimide-DOTA and Tb were site-specifically conjugated to the ligand. To assess the chelation efficiency, the eFGF1-^161^Tb product was separated by thin-layer chromatography and visualized by phosphor imaging. Free ^161^Tb^3+^ cation is positively charged and retained in the stationary phase; however, when it was conjugated to eFGF1-DOTA, it moved together with the mobile phase. As shown in Figure 4c, the conjugation efficiency of eFGF1-^161^Tb was above 90%.

Native eFGF1 does not show fluorescence around 350 nm because the tryptophan residue is quenched by surrounding amino acid groups, while unfolded eFGF1 shows a fluorescence peak at 350 nm.^30^Figure S5 shows the eFGF1 protein fluorescence spectrum after each production step and suggests that eFGF1 unfolded in pH6.0 buffer during the chelation step but folded back when exchanged to pH7.4 buffer.

To further check the target binding ability of eFGF1-Tb, we treated MCF-7 FGFR1 cells with various concentrations of wild-type FGF1, eFGF1, and eFGF1-Tb for 15 min and examined the activation of the downstream signaling of FGFR1. The Western blot results in Figure S6 show that eFGF1-Tb conjugates stimulate the activation of FGFR1 (pFGFR1), PLCγ, and ERK equally well as wild-type FGF1 and eFGF1, indicating that the conjugation process does not affect the binding ability of eFGF1.

### Surface Binding and Intracellular Uptake of eFGF1-^161^Tb on FGFR-Overexpressing Cells

**Figure 5:**
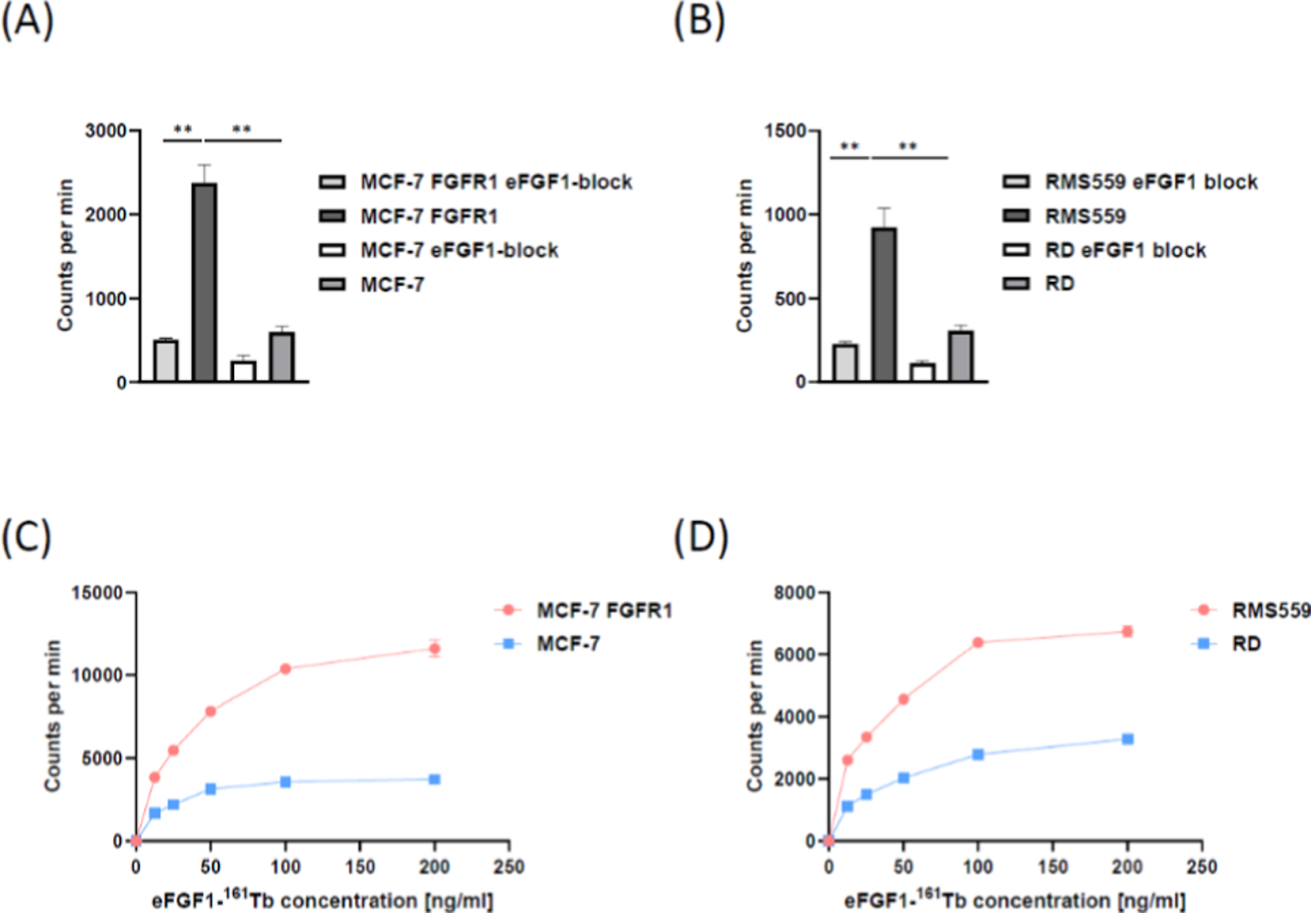
Receptor binding and cellular uptake of eFGF1-^161^Tb conjugates. (A) Binding of eFGF1-^161^Tb conjugates to FGF receptors in the MCF-7 FGFR1 cell line. MCF-7 serves as a negative control. (B) Binding of eFGF1-^161^Tb conjugates to naturally FGFR overexpressed cell line, RMS559. RD serves as a negative control. Twelve million cells were preblocked with 10 μg eFGF1 ligand and then incubated with 5 ng radioligand for 1 h. For (A,B), data are represented as mean values ± SD (from *n* = 3 independent experimental replicates). Significance in (A,B) was tested using a two-tailed, unpaired *t*-test and is indicated as **P < 0.01. (C,D) Uptake of eFGF1-^161^Tb conjugates in MCF-7 FGFR1/MCF-7, and RMS559/RD, after 1 h incubation with 12.5, 25, 50, 100, and 200 ng/mL eFGF1-^161^Tb radioligands. For (C,D), data are represented as mean values ± SD (from *n* = 3 independent experimental replicates).

The binding of eFGF1-^161^Tb radioligands to FGFR receptors was evaluated by incubation of 5 ng radioconjugates (protein amount) with 12 million MCF-7 FGFR1 and RMS559 cells at 37 °C for 1 h. After washing away the unbound eFGF1-^161^Tb, the cells were measured with a gamma counter. As shown in Figure 5A,B, MCF-7 FGFR1 cells bind 5 times more eFGF1-^161^Tb than MCF-7 cells, and RMS559 cells bind 3 times more than RD cells. When the cells were pretreated with 10 μg unlabeled eFGF1 ligand, binding between radioligands and receptors on both MCF-7 FGFR1 and RMS559 could be significantly outcompeted. This indicates that the binding of eFGF1-^161^Tb to the cells is specific and receptor-mediated.

Internalization of radioligands into the cells was assessed by incubating different concentrations of eFGF1-^161^Tb with cells at 37 °C for 2 h and then washing away the surface-bound radioligands with high salt, low pH buffer (Figure 5C,D). The signal intensity of internalized radioligands showed a dose-dependent increase with higher eFGF1 concentrations. Both MCF-7 FGFR1 and RMS559 cells internalized 4 times more eFGF1-^161^Tb than MCF-7 and RD cells, which indicates that eFGF1 as a vector can deliver more radionuclides into FGFR overexpressing cell lines.

Previous reports have shown that extracellular FGF1 can be translocated to the nuclear fraction of cells after binding to its receptor.^31−33^ As ^161^Tb emits Auger electrons whose impact should be more pronounced when the radionuclides are close to the nucleus due to the short radiation length, we investigated the nuclear targeting of eFGF1-^161^Tb. Interestingly, around 10% of the radioligand was recovered from the nuclear fraction after careful fractionation of the cells (Figure S10). Several inhibitors of FGF1 nuclear translocation have been reported,^34−36^ and we also included these in our tests. As can be seen in Figure S10, when cells were pretreated with 100 μM bafilomycin A1, 10 nM radicicol, or 10 μM SB203590, the intensity of the eFGF1-^161^Tb signal was severely decreased, demonstrating that the signal in the nuclear fraction was specific.

### Cancer Cell–Suppressive Activity of eFGF1-^161^Tb

**Figure 6:**
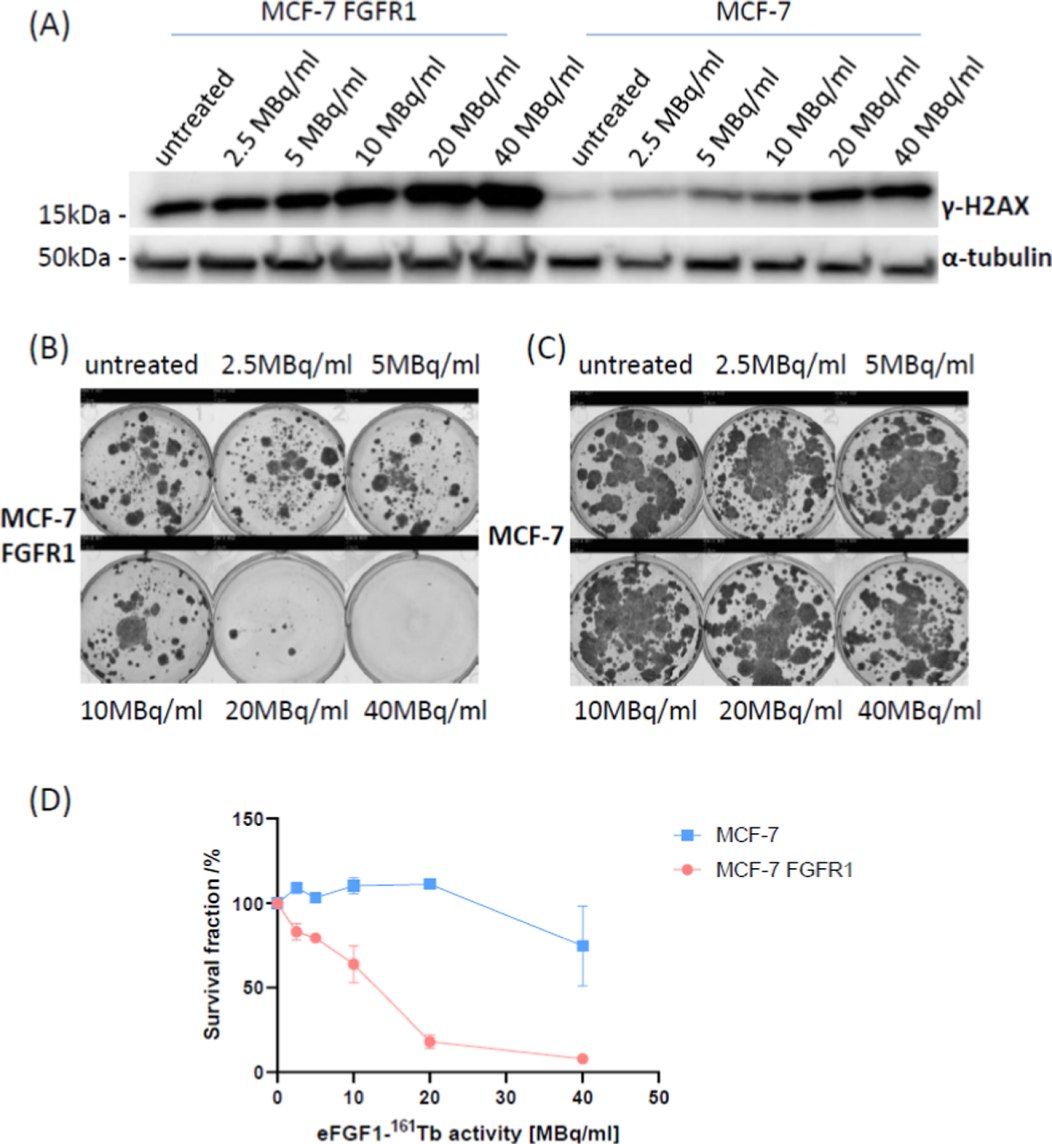
Cytotoxicity of eFGF1-^161^Tb radioligands in breast cancer cells. (A) Western blot of γ-H2AX expression in MCF-7 FGFR1 and MCF-7 cells after treatment for 24 h. α-tubulin serves as the internal control. (B,C) Representative colony images of MCF-7 and MCF-7 FGFR1 after treatment with of 2.5, 5, 10, 20, and 40 MBq/mL eFGF1-^161^Tb radioligands for 2 h. (D) Cell survival fraction rate of MCF-7 and MCF-7 FGFR1 cells, which was measured by clonogenic assay. Data is represented as mean values ± SD (from *n* = 2 independent experimental replicates).

To investigate the cytotoxicity of eFGF1-^161^Tb, MCF-7 FGFR1 and MCF-7 cells were incubated with different activities of eFGF1-^161^Tb radioligands, followed by Western blot to assess the expression of γ-H2AX, a marker of DNA double-strand breaks.^37^ As shown in Figure 6A, γ-H2AX expression in MCF-7 FGFR1 cells was significantly higher than that in MCF-7 cells after treatment with similar doses of eFGF1-^161^Tb radioligands, and the band of γ-H2AX increased in a concentration-dependent manner.

To further evaluate the inhibition of eFGF1-^161^Tb on tumor cells, MCF-7 FGFR1 and MCF-7 cells were treated with various activities of eFGF1-^161^Tb for 2 h, seeded with an equal number in 6-well plates, and kept for 3 weeks to form colonies. Compared to the untreated group, the survival and proliferation of MCF-7 FGFR1 were severely inhibited by radionuclides (Figure 6B,C). Figure 6D shows the survival fraction of each cell line after treatment with different activities of radioligands. 40 MBq/mL eFGF1-^161^Tb reduced the cell survival on both MCF-7 FGFR1 and MCF-7, while the cell inhibition rate on MCF-7 FGFR1 is 92%, but only 20% in MCF-7. Lower doses of radioligand treatment did not inhibit the proliferation of MCF-7, while MCF-7 FGFR1 cells were much more sensitive to the radioligands, 20 MBq/mL eFGF1-^161^Tb treatments could inhibit nearly 80% cell proliferation, and 2.5 MBq/mL could reduce the cell survival rate by 20%.

**Figure 7:**
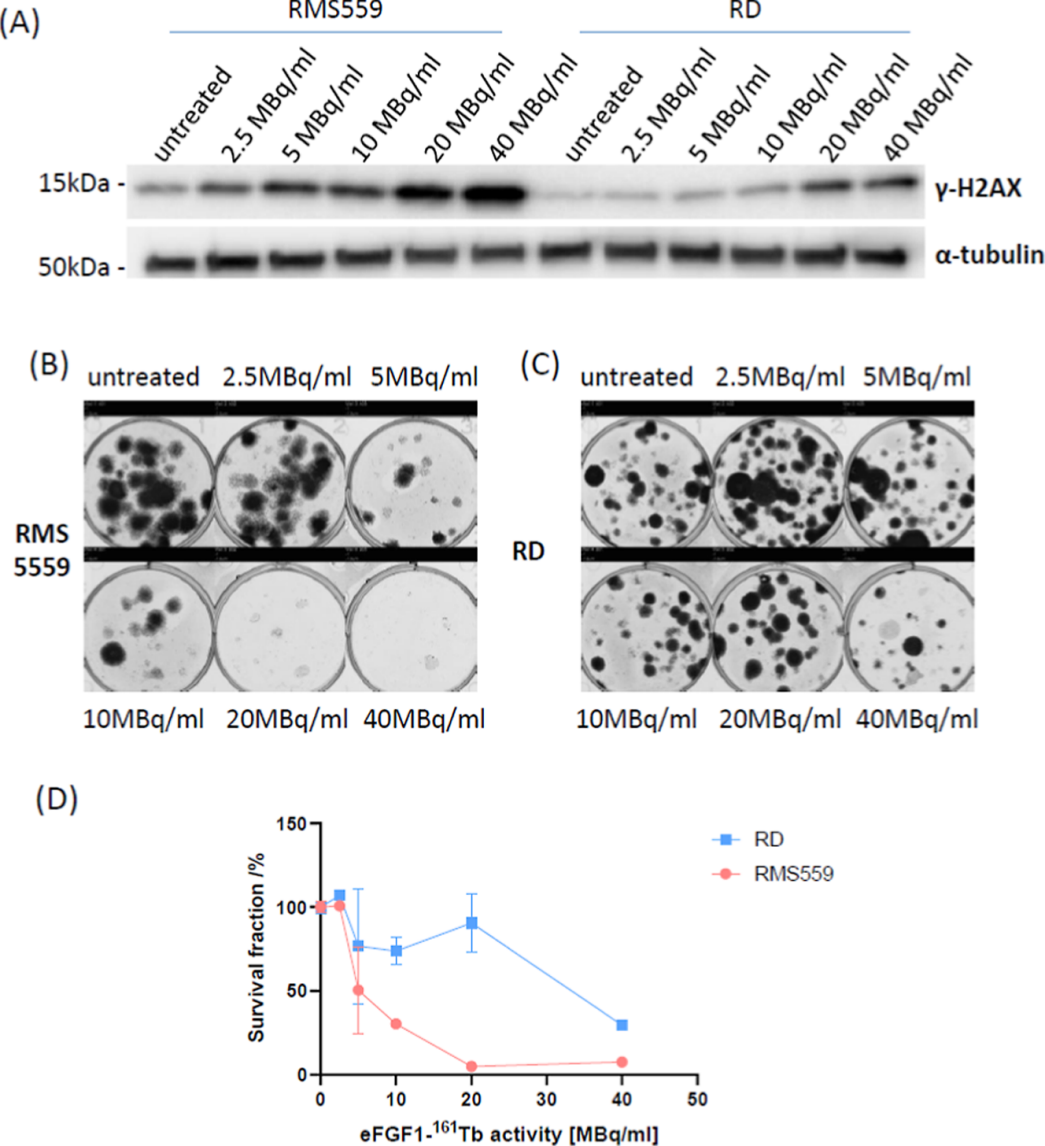
Cytotoxicity of eFGF1-^161^Tb radioligands in the naturally FGFR overexpression rhabdomyosarcoma cells. (A) Western blot of γ-H2AX expression in RMS559 and RD cells after treatment with eFGF1-^161^Tb activity from 2.5, 5, 10, 20, and 40 MBq/mL for 24 h. α-tubulin serves as the internal control. (B,C) Representative image of clonogenic assays of RD and RMS559 after treatment with 2.5, 5, 10, 20, and 40 MBq/mL eFGF1-^161^Tb radioligands for 2 h. (D) Cell survival fraction rate of RMS559 and RD, which was measured by the clonogenic assay. Data is represented as mean values ± SD (from *n* = 2 independent experimental replicates).

We then further assessed the tumor suppressive activity of eFGF1-^161^Tb on the naturally FGFR overexpressing cell line RMS559. Figure 7A shows that the expression level of γ-H2AX in RMS559 is higher than that in the negative control RD cells after treatment with the radioligands. The clonogenic assays presented in Figure 7B,D suggest that the conjugate is significantly more effective in RMS559 cells than in RD cells. Similar results were also found using spheroids (Figure S11). These results suggest that eFGF1 can deliver more ^161^Tb to cells and cause stronger damage to DNA to inhibit the proliferation of FGFR overexpressing tumor cells.

### Doxorubicin Delivered by eFGF1 for the Targeting of Rhabdomyosarcoma Cells

**Figure 8:**
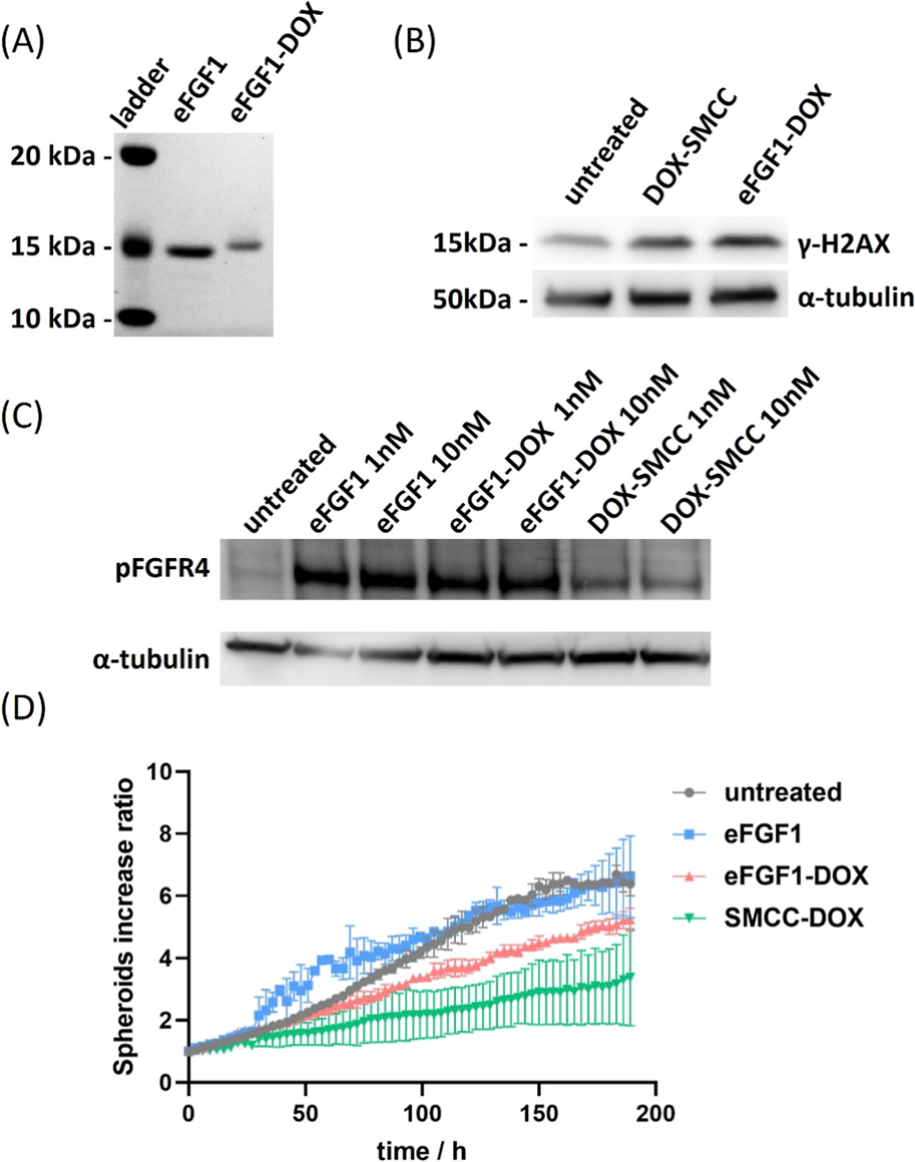
Cytotoxicity of eFGF1-DOX conjugates in FGFR overexpression cells. (A) Molecular weight increases of eFGF1-DOX conjugates on PAGE gel. (B) Western blot of γ-H2AX expression in RMS559 cells after treatment with 10 μM DOX-SMCC and eFGF1-DOX for 24 h. α-tubulin serves as the internal control. (C) Activation of signaling in RMS559 cells after stimulation with 1 nM or 10 nM eFGF1, DOX-SMCC, and eFGF1-DOX conjugates. (D) Increase ratio of RMS559 spheroids after treatment with 20 μM eFGF1, DOX-SMCC, and eFGF1-DOX. Data is represented as mean values ± SD (from *n* = 2 independent experimental replicates).

Besides radionuclides, we also evaluated the possibility of eFGF1 to deliver the chemotherapeutic drug doxorubicin (DOX) to FGFR-overexpressing cancer cells. With a free maleimide functional group, the commercially available compound DOX-SMCC can be conjugated to the free thiol on the C-terminus of eFGF1. The conjugation efficiency was confirmed by the molecular weight increase visible in electrophoresis (Figure 8A). We first wanted to test whether this construct could specifically target cells overexpressing FGFRs. We took advantage of the inherent fluorescent properties of DOX and used confocal microscopy to observe whether eFGF1-DOX was associated with cells overexpressing FGFR1. Indeed, eFGF1-DOX was efficiently internalized in MCF-7 FGFR1 cells, while very little eFGF1-DOX was taken up by MCF-7 cells with low amounts of receptor (Figure S12). This clearly indicates that eFGF1-DOX targets only FGFR1 overexpressing cells. After treatment with 10 μM DOX or eFGF1-DOX for 24 h, the level of γ-H2AX in RMS559 was assessed by Western blot. As shown in Figure 8B, both eFGF1-DOX and DOX induced higher levels of γ-H2AX. The Western blot presented in Figure 8C demonstrated that eFGF1-DOX can activate FGFR4 similarly as FGF1, indicating that conjugation with DOX does not affect the binding between eFGF1 ligand and FGFR4. To study the cytotoxicity of eFGF1-DOX, RMS559 cells were seeded in ultralow attachment plates and cultured for 3 days and then treated with eFGF1, DOX-SMCC, and eFGF1-DOX for another 7 days. Spheroids in each group were imaged by Incucyte S3 every 3 h, and their initial size was normalized to the untreated group. eFGF1 does not cause inhibition on spheroid growth, while the eFGF1-DOX and unconjugated DOX inhibit the growth of RMS559 spheroids at 10–20 μM concentration (Figures 8D and S13). These results suggested that eFGF1 can also serve as a targeting vector for chemotherapeutic drugs.

## Discussion

Targeted radionuclide therapy shows many advantages in cancer therapy by delivering radionuclides directly to cancer cells, aiming to enhance radiotherapy efficiency and avoid side effects. ^161^Tb has been highlighted as an alternative to the clinical radionuclide ^177^Lu because of its coemission of conversion and Auger electrons. In this study, we generated a stable FGF1 with only one free thiol in the C-terminus, allowing site-specific conjugation with ^161^Tb for targeting FGFR overexpressed cancers.

FGF1, which is a member of the FGF family, can be recognized and internalized by all FGF receptors. However, the low thermodynamic stability and short half-life of native FGF1 limit its application in protein therapeutic research.^38^ Here, we confirm and extend previous studies showing that the engineered FGF1 vector can keep structural integrity at 37 °C for more than 10 days after mutating three amino acids, Q40P/S47I/H93G.^26^ Even though antibody conjugates are quite promising in targeted cancer therapy, most of them are generated with traditional methods that result in heterogeneous mixtures and low reproducibility.^39,40^ To construct a site-specific labeling vector, we added glycine and cysteine at the C-terminus of FGF1 and replaced three cysteines with serines. With the free thiol, the engineered FGF1 vector could easily be conjugated to other molecules on the C-terminus, including fluorophores, DOTA-^161^Tb and doxorubicin.

Previous studies have shown that amino acid mutations might affect the binding and targeting function of proteins,^41^ so we checked the eFGF1 targeting function by structural investigations, confocal microscopy, flow cytometry, and Western blot. eFGF1 displays the same activation of downstream signaling pathways of FGFR as wild-type FGF1 and significant internalization in FGFR-overexpressing cells. Moreover, the eFGF1 vector shows better infiltration into 3D tumor spheroids than an FGFR antibody. The FGFR antibody is a type of IgG protein, with a molecular weight of 150 kDa, while eFGF1 is only 15.3 kDa, more similar to a nanobody. It was previously reported that proteins with smaller sizes have advantages for tumor penetration,^42^ so the eFGF1 ligand could have a benefit in treatment of solid tumors.

Even though a previous study suggested FGF1 is stable only in neutral pH,^43^ we surprisingly found that although the engineered FGF1 unfolds in pH 6.0, the protein could fold back when exchanged to pH 7.4 and remained able to bind FGFR. Because maleimide-DOTA was site-specifically labeled on eFGF1 and only one cation can be chelated on the macrocyclic ring of DOTA, ^161^Tb could be conjugated to the eFGF1 vector with high chelation efficiency above 90% and then be effectively internalized into FGFR overexpressed cells. A previous study showed that FGF1 could be internalized into cells within 2 h.^44^ Therefore, we treated cells with eFGF1-^161^Tb for 2 h, and eFGF1-^161^Tb showed high binding selectivity for FGFR overexpressing cells, which also resulted in a higher cytotoxicity in the targeted cells. This suggests that our developed eFGF1-^161^Tb could be a promising candidate in targeted radionuclide cancer therapy. The efficient and specific targeting could potentially reduce side effects if used in cancer therapy.

Doxorubicin is a chemotherapeutic drug that is used for the treatment of various cancers. However, the severe side effects limit its clinical application.^45^ We found that eFGF1 could also be conjugated to doxorubicin and deliver the drug into the cells. Taken together, the engineered FGF1 vector is highly versatile and could be extended to deliver other potent agents.

## Experimental Procedures

Caution! The isotope ^161^Tb (half-life = 6.9 days) emits low-energy β and γ particles as well as conversion and Auger electrons, posing a significant health hazard. All experimental procedures involving ^161^Tb were carried out in facilities specially designed and approved for handling radioactive materials. Proper radiation safety protocols, including the use of appropriate shielding and personal protective equipment, were adhered to throughout the study.

### Chemicals and Radionuclides

All chemicals (analytical grade; ultrapure for radiolabeling), TLC Silica gel 60 F_254_, and solvents (HPLC-grade; metal-free for radiolabeling) were purchased from Merck. A 96-well ultralow attachment round-bottom plate (CLS7007) was ordered from Corning. Maleimido-monoamide-DOTA (B-272) was purchased from Macrocyclics. Doxorubicin (DOX)-SMCC (HY-116063) was purchased from MedChemExpress. The antibodies used were as follows: rabbit anti-FGFR1 (#9740), rabbit antiphospho-FGFR (Tyr653/654) (#3471), mouse antiphospho-ERK 1/2 (pERK 1/2, Thr202/Tyr204) (#9106), and rabbit antiphospho-PLCγ (pPLCγ, Tyr783) (#14008) from Cell Signaling Technology; mouse anti-α-Tubulin (CP06) from Calbiochem; mouse anti-FGF1 (SAB1403812) from Merck; mouse anti-γH2AX (05-636) from Millipore; and Human Phospho-FGFR1-4 (Y653/Y654) (#3285) antibody from biotechne. DL550 Maleimide, DL633 NHS ester, fluorescent dye removal column (Product no. 22858), Zeba Spin Desalting Columns (89882), Hoechst 33342 (H3570), and ProLong Diamond Antifade Mounting (P36961) were purchased from Thermo Fisher. The monoclonal FGFR1 antibody was generated by GenScript.

### Preparation of [^161^Tb]TbCl^3^

No-carrier-added [^161^Tb]TbCl^3^ (^161^Tb in abbreviation) (^161^TbCl_3_ in 0.1 M HCl) was prepared in-house at the Czech Technical University in Prague, Faculty of Nuclear Sciences and Physical Engineering, Prague, Czech Republic. The highly enriched ^160^Gd material was used (98.2% enrichment, oxide form, Isoflex, USA) as a target material for neutron irradiation in an experimental nuclear reactor LVR-15 (CV Rez s.r.o., Czech Republic) operated at 9.68 MW_t_ power by employing the ^160^Gd(*n*,γ)^161^Gd → ^161^Tb reaction sequence. Typically, some 50 mg of [^160^Gd] Gd_2_O_3_ was flame-sealed in a quartz ampule, placed in an aluminum container, and irradiated at 6–7 × 10^13^ n.cm^2^ s^–1^ thermal neutron flux at H5/H6 irradiation channels for 12 days, providing some 12 GBq of ^161^Tb at the end of irradiation. Further, the target was dissolved in 0.5 mL of concentrated nitric acid, evaporated to dryness, and redissolved in ultrapure water (18 MΩ). Gadolinium and terbium separation was performed using the 2-hydroxybutyric acid/Dowex 50 method, similar to Lehenberger [21]. Shortly, the dissolved target was loaded on a Dowex 50 × 8 converted to NH_4_^+^ cycle, 200–400 mesh (Merck, Czech) column, bed volume of 3 mL. The ^161^Tb was eluted with 0.13 M 2-hydroxybutyric acid solution (pH set to 4.5), and the ^160^Gd with 0.5 M 2-hydroxybutyric acid solution for further recycling. Terbium was converted to chloride form by postcolumn purification from 2-hydroxybutyric acid on a 0.5 mL Dowex 50 × 8 cartridge with 4 M HCl and subsequent evaporation and final reformulation into 0.1 M HCl. High radionuclidic purity > 99,999% (only radioactive impurity detected ^160^Tb) and specific activity over 3 GBq/μg (related to stable Tb) were determined for the final ^161^Tb sample. A sample of about 3.5 GBq of the ^161^Tb was shipped for labeling within 1 week after separation.

### Plasmid Preparation

To make the construct encoding the FGF1-based vector, we ordered a gBlock DNA fragment (Integrated DNA Technologies, Inc.) containing the desired changes in the structure (see Figure S2). The fragment was made with overhanging restriction enzyme sites for easy cloning into the pET-21d vector. The vector and fragment were cut with NcoI and HindIII (NEB), bands gel-purified, and ligated together overnight using T4 DNA ligase at 16 °C (NEB). The construct was sequenced to verify the correct structure of the resulting plasmid (pET21d-FGF1-GC).

### Engineered FGF1 Production

Production of recombinant eFGF1 was performed, as described previously.^16^ The pET-21d-FGF1-GC plasmid was transformed into the *E. coli* BL21 (DE3) pLysS expression strain from Merck Biosciences to express recombinant protein (eFGF1). Bacteria were grown to OD_600_ = 0.9 in LB medium with 100 μg/mL ampicillin at 37 °C and a shaking speed of 250 rpm. Then, the temperature was decreased to 25 °C, and protein production was induced by adding IPTG to a final concentration of 0.5 mM and continued for 12 h. After this time, bacteria were harvested by centrifugation at 3000*g* for 20 min and washed with PBS once, then resuspended in buffer A (20 mM Tris, 0.5 M NaCl, 1 mM dithiothreitol (DTT), 1 mM EDTA, 0.1 mM PMSF, pH 7.4) and sonicated for 5 min for 4 rounds. The cell debris was separated by ultracentrifugation at 20,000*g* at 4 °C for 45 min. The clarified cell lysate was loaded onto a heparin-sepharose gravity column. After 1 h incubation at 4 °C, the column was washed with buffer B (20 mM Tris, 0.7 M NaCl, 1 mM dithiothreitol (DTT), 1 mM EDTA, 0.1 mM PMSF, pH 7.4) for 4 column volume. Protein was eluted with buffer C (20 mM Tris, 2 M NaCl, 1 mM dithiothreitol (DTT), 1 mM EDTA, and 0.1 mM PMSF, pH 7.4) and quantified by Nanodrop.

### Cell Culture

The MCF-7 breast cancer cell line was kindly provided by Prof. Dr. Harald Stenmark’s group, Oslo University Hospital. The MCF-7 cells stably expressing FGFR1 (MCF-7 FGFR1) were kindly provided by Dr. Ellen M. Haugsten. Both cell lines were grown in RPMI-1640 complete media containing 10% fetal calf serum and 100 U/mL penicillin–streptomycin. The RMS559 cell line was a kind gift from Prof. Jonathan Fletcher, and GFP-RMS559 stably expressing GFP was kindly provided by Else Munthe. Both cell lines were grown in Iscove’s Modified Dulbecco medium supplemented with 15% fetal calf serum and 100 U/mL penicillin–streptomycin. The RD (CCL-136) cell line was obtained from ATCC, which was cultured in DMEM media with 10% fetal calf serum and 100 U/mL penicillin–streptomycin. The cell lines were tested negative for mycoplasma contamination by PCR.

### Fluorophore Labeling eFGF1

DL550 maleimide and DL633 NHS ester were dissolved in DMSO with a final concentration of 5 mg/mL. To label eFGF1 with DL550 or DL633 fluorophore, FGF1 was exchanged into PBS (pH7.4) buffer, mixed with dye at a ratio of 1:10, and incubated at room temperature for 1 h. Excess fluorophore was washed away with fluorescent dye removal columns. Labeling of FGF1 was confirmed by gel electrophoresis and kept at −20 °C for storage. To label FGFR1 antibody with DL633 NHS ester, FGFR1 antibody was incubated with DL633 fluorophore with a ratio of 1:20 at room temperature for 1 h. Excess dye was removed with a fluorescent dye removal column. Final conjugates were confirmed by gel electrophoresis and stored at −20 °C.

### Confocal Microscopy

MCF-7, MCF-7 FGFR1, RD, and RMS559 cells were seeded on coverslips in 24-well plates with 1 × 10^5^ cells per well. After 24 h of incubation, cells were treated with DL550-labeled eFGF1 at a concentration of 10, 20, 50, 100, and 200 ng/mL at 37 °C for 2 h in complete media containing 10 U/mL heparin sulfate. Cells were washed with PBS and fixed in 4% formaldehyde (Sigma-Aldrich, HT5012) at room temperature for 15 min. After washing with PBS three times, cells were stained with Hoechst 33342 for 5 min at room temperature. The coverslips were immobilized on microscope slides with ProLong Diamond Antifade Mounting and dried in the dark at 4 °C overnight before imaging. Images were captured with an ×63 objective on a Zeiss confocal Laser Scanning Microscope (LSM) 880 (Jena, Germany) and prepared with ZEN software.

### Flow Cytometry Analysis

MCF-7, MCF-7 FGFR1, RD, and RMS559 cells were seeded in 6-well plates with 3 × 10^5^ cells per well. After 24 h of incubation, cells were treated with DL550-labeled eFGF1 with the same concentrations as above at 37 °C for 2 h. Cells were detached with trypsin, washed with PBS buffer three times, and then measured with a BD LSR II flow cytometer. Data was plotted with Flowjo 10.10 software.

### Confocal Microscopy of Spheroids

Spheroids were formed by seeding 500 GFP-RMS559 cells in 100 μL of culture medium per well in a 96-well ultralow attachment round-bottom plate. The plates were centrifuged at 1000*g* for 5 min and then incubated at 37 °C for 3 days for the formation of spheroids. Each well was treated with 5 μg/mL of eFGF1- DL633 or 10 μg/mL of Ab- DL633 for 24 h. Cells were stained with Hoechst 33342 for 16 h before imaging. All the images were captured with an ×20 objective on a Zeiss confocal LSM 880 (Jena, Germany) and processed with Imaris software.

### ^161^Tb Radiolabeling and Quality Control

Maleimido-monoamide-DOTA was solubilized in DMSO with a final concentration of 100 mM. To conjugate with maleimido-DOTA, 238 μg of eFGF1 protein was exchanged into 100 μL of 0.5 M NaOAc (sodium acetate) pH 7.4 buffer (2.38 mg/mL, 150 μM) and mixed with 1.5 μL of 100 mM maleimido-DOTA and then kept at room temperature for 1 h. Excess maleimido-DOTA was washed away with Zeba Spin Desalting Columns. The final eFGF1-DOTA product was stored in a freezer before chelating with ^161^Tb. 92 μL 2.55 GBq ^161^TbCl_3_ (in 0.1 M HCl) was added to 92 μL 0.5 M NaOAc pH 7.4 buffer to adjust the pH to 6.0, with a final concentration of 13.85 GBq/mL (20 μM, with the decay coefficient constant of 0.7293). eFGF1-DOTA conjugates in 0.5 M NaOAc pH 6.0 buffer were mixed with the above ^161^Tb solution with a ratio of 10:1 and incubated at 37 °C for 16 h. To terminate the chelation, 0.5 M EDTA pH 8.0 was added into the mixture with 5 min incubation at room temperature. One M NaOH was added to the mixture to adjust pH to 7.4. Final chelation efficiency was measured by thin layer chromatography, identified with a labeling efficiency of 90.2%. Free ^161^Tb^3+^ and eFGF1-^161^Tb were normalized to have equal radioactivity, a drop (1 μL) of each sample was applied to a TLC Silica gel, and the sample was separated with the PBS mobile phase for 40 min. The plate was dried at 37 °C for 5 min and then exposed in a phosphor screen cassette for 3 min. Images were captured by Azure biosystems in the phosphor mode. Quality control of the final eFGF1-DOTA-Tb conjugates was performed using the nonradioactive Tb isotope (TbCl_3_) with 16% PAGE gel electrophoresis and MAXIS II LC Q-TOF mass spectrometry.

### eFGF1-Tb Targeting Function

To check the proper folding of eFGF1-DOTA-Tb, we monitored the fluorescence of the single tryptophan residue in FGF1, which remains quenched in the native conformation and reappears under denaturation with a peak at 353 nm.^46^ Solutions of FGF1, FGF1-DOTA, and FGF1-DOTA-Tb in 0.5 M NaOAc pH 6.0 and FGF1-DOTA-Tb in 0.5 M NaOAc pH 7.4 were diluted into 10 μM with the corresponding buffer, then measured by fluorescence spectroscopy at 25 °C and a 10 mm quartz cuvette, with excitation at 280 nm and emission spectra from 300 to 450 nm. The final spectrum was plotted with the GraphPad Prism software.

### Binding of eFGF1-^161^Tb Radioligands to FGF Receptors

To confirm the binding ability of eFGF1-^161^Tb radioligands with FGFR receptors, MCF-7 FGFR1 cells were seeded in 12-well plates 1 day before treatment, 2 × 10^5^ cells per well. Cells were treated with wild-type FGF1, eFGF1, and cold (nonradioactive) eFGF1-Tb with concentrations of 1, 2, 10, 20, 100, and 200 ng/mL for 15 min in complete media containing 10 U/mL heparin. After being washed with cold PBS three times, cells were lysed with 2× sample buffer (Bio-Rad). Lysate was loaded on 4–20% gradient gels (Bio-Rad) and then transferred on a PVDF membrane. Membranes were then incubated with indicated primary antibodies with 1:1000 dilutions, followed by corresponding secondary antibodies coupled to HRP. Images were captured on a Biorad ChemDoc imaging system.

To check the binding ability of radiolabeled eFGF1-^161^Tb with FGF receptors, MCF-7, MCF-7 FGFR1, RD, and RMS559 cells were detached with 1 mM EDTA in PBS buffer at room temperature for 3 min and washed with PBS w/0.5% BSA in PBS buffer three times. All of the cell pellets were normalized to the same concentration of 6 × 10^7^/mL. Aliquots of 200 μL of each cell suspension (12 million cells) were added to the counting tubes and incubated with 5 ng eFGF1-^161^Tb at 37 °C and a shaking of 500 rpm for 1 h. For FGF1 blocking control, cells were pre-incubated with 10 μg of eFGF1 at 37 °C for 15 min. After incubation, cells were washed with PBS w/0.5% BSA three times by centrifugation at 500*g* for 3 min. Radioactivity in the samples was measured with a HIDEX Gamma counter.

### Uptake of eFGF1-^161^Tb

MCF-7, MCF-7 FGFR1, RD, and RMS559 cells were seeded in 12-well plates, 2 × 10^5^ cells per well. After 1 day of incubation, cells were treated with eFGF1-^161^Tb with concentrations of 12.5, 25, 50, 100, and 200 ng/mL (eFGF1 concentration) at 37 °C for 2 h in complete media containing 10 U/mL heparin. Unbound conjugates were washed away with HSLP (2 M NaCl, 20 mM NaOAc, pH4.0) buffer three times. After lysis with 1 M KOH, internalized eFGF1-^161^Tb radioactivity was measured with a HIDEX Gamma counter.

### WB of γ-H2AX

MCF-7, MCF-7 FGFR1, RD, and RMS559 cells were seeded in 12 well plates with 2 × 10^5^cells per well. After 1 day of incubation, cells were treated with eFGF1-^161^Tb with an activity of 2.5, 5, 10, 20, and 40 MBq/mL at 37 °C for 24 h in complete media containing 10 U/mL heparin. Then, cells were washed with HSLP buffer twice, PBS buffer once, and lysed with 80 μL 2× Laemmli buffer (Biorad). Lysate was loaded on 4–20% gradient gels (Bio-Rad) and then transferred on the PVDF membrane. Membranes were then incubated with γH2AX primary antibodies with 1:200 dilutions, followed by corresponding secondary antibodies coupled to HRP. Images were captured on the Biorad ChemDoc imaging system.

### Cytotoxicity of eFGF1-^161^Tb

MCF-7, MCF-7 FGFR1, RD, and RMS559 cells were detached with 1 mM EDTA in PBS buffer at room temperature for 3 min, and the reaction was terminated with complete media and washed with PBS buffer three times. All the cell pellets were normalized to the same concentration of 2 × 10^5^/mL in complete media containing 10 U/mL heparin. Aliquots of 1 mL of each cell suspension (0.2 million cells) were added to EP tubes and incubated with eFGF1-^161^Tb with activities of 2.5, 5, 10, 20, and 40 MBq/mL at 37 °C and a stirring speed of 500 rpm for 2 h. After incubation, cells were washed with PBS three times by centrifugation at 500*g* for 3 min and finally resuspended in complete media.

To perform the clonogenic assay, treated cells were seeded in a 6-well plate, 1000 cells per well, and then kept for 4 weeks by changing media once per week. The cytotoxicity of eFGF1-^161^Tb was assessed by crystal violet staining. After 4 weeks of culture, colonies were fixed in 4% formaldehyde at room temperature for 15 min, incubated with 0.2% crystal violet (Sigma, V5265) at room temperature for 1 h, washed with PBS three times, and dried at room temperature for 1 h. Colony images were captured by a GelCount (OXFORD OPTRONIX). To assess the survival fraction of eFGF1-^161^Tb, stained colonies were dissolved in 10% acetic acid and measured by a plate reader at an absorption of 590 nm (PerkinElmer Victor X3).

To generate spheroids with irradiated cells, a cell suspension of 500 cells in 100 μL was seeded in a 96-well ultralow attachment round-bottom plate, followed by centrifugation at 1000*g* for 5 min. Spheroid sizes and morphologies were captured once per week by the Incucyte S3 spheroids mode.

### eFGF1-DOX Generation

DOX-SMCC was dissolved in DMSO with a final concentration of 5 mg/mL. eFGF1 was exchanged into PBS buffer (pH7.4) before conjugation. Mix eFGF1 was mixed with DOX-SMCC at a ratio of 1:10 and incubated at room temperature for 1 h. Excess DOX-SMCC was washed away with Zeba Spin Desalting Columns. eFGF1-DOX conjugates were confirmed by gel electrophoresis and kept at −20 °C for storage.

### eFGF1-DOX Targeting Function

The targeting ability of eFGF1-DOX conjugates was confirmed by Western blot. RMS559 cells were seeded in 12-well plates 1 day before treatment, 2 × 10^5^ cells per well. Cells were treated with eFGF1, eFGF1-DOX, and DOX-SMCC with concentrations of 1 and 10 nM for 15 min in complete media containing 10 U/mL heparin. After washing with cold PBS three times, cells were lysed with 2 x sample buffer (Bio-Rad). Lysate was loaded on 4–20% gradient gels (Bio-Rad) and then transferred on the PVDF membrane. Membranes were then incubated with human phospho-FGFR1-4 (Y653/Y654) primary antibodies with 1:500 dilutions, followed by corresponding secondary antibodies coupled to HRP. Images were captured on a Biorad ChemDoc imaging system.

### Cytotoxicity of eFGF1-DOX

RD and RMS559 cells were seeded in 12 well plates, 2 × 10^5^cells per well. After 1 day of incubation, cells were treated with 10 μM DOX or eFGF1-DOX at 37 °C for 24 h in complete media containing 10 U/mL heparin. Then, cells were washed with HSLP buffer twice and PBS buffer once and lysed with 80 μL of 2x loading buffer (Biorad). Lysate was loaded on 4–20% gradient gels (Bio-Rad) and then transferred on the PVDF membrane. Membranes were then incubated with the γ-H2AX primary antibody, followed by corresponding secondary antibodies coupled to HRP. Images were captured on a Biorad ChemDoc imaging system.

To evaluate inhibition of eFGF1-DOX on cell proliferation, we generate RMS559 spheroids by seeding 500 cells in a 96-well ultralow attachment round-bottom plate, followed with centrifugation at 1000*g* for 5 min. After 3 days, spheroids were treated with eFGF1, eFGF1-DOX, and DOX, respectively, at concentrations of 5, 10, and 20 μM. Images of spheroids in each group were captured by Incucyte S3 every 3 h for another 7 days. Spheroid sizes were normalized to the untreated group and plotted with GraphPad Prism software.

### Uptake of eFGF1-DOX

MCF-7 and MCF-7 FGFR1 cells were seeded on coverslips in 24-well plates with 1 × 10^5^ cells per well. After 24 h incubation, cells were treated with eFGF1-DOX at concentrations of 1 and 10 μM at 37 °C for 2 h in complete media containing 10 U/mL heparin sulfate. Cells were washed with PBS and fixed in 4% formaldehyde (Sigma-Aldrich, HT5012) at room temperature for 15 min. After washing with PBS three times, cells were stained with Hoechst 33342 for 5 min at room temperature. The coverslips were immobilized on microscope slides with ProLong Diamond Antifade Mounting and dried in the dark at 4 °C overnight before imaging. Images were captured with the ×63 objective on a Zeiss confocal LSM 880 (Jena, Germany) and prepared with ZEN software.

### Structural Model of Mutated FGF1 and of the Dimeric 2:2:2 FGF1: FGFR1: Heparin Ternary Complex

An AlphaFold3 (AF3) model of mutated and N- and C-terminally modified FGF1 was generated with the AlphaFold Server web service.^47^ The AF3 per-residue confidence metric pLDDT values^48^ and structural disorder predictions with DISOPRED3^49^ suggest that the N-and C-termini of human FGF1 are structurally disordered, while the main core segment is folded and mainly structurally ordered. The Protein Data Bank entry 1EVT^50^ contains the experimental crystal structure of the FGF1: FGFR1 complex monomer. The structurally ordered part of the AF3 model (residues Lys9 to Ser138) is highly similar to the corresponding part of the FGF1 crystal structure in the complex structure (RMSD = 0.49 Å), suggesting both that the FGF1 AF3 model is accurate and that the FGF1 core structure is not affected significantly by FGFR binding.

The backbone conformation of the N-terminal, structurally disordered part of the AF3 model was manually altered to avoid collision with FGFR1 when the model was docked into the FGF binding site by structurally aligning it with FGF in the experimental FGF: FGFR complex structures. The resulting FGF1 model is shown in Figure S1.

The X-ray structure of 1FQ9 contains the dimeric 2:2:2 FGF2: FGFR1: heparin ternary complex. In order to build an FGF1: FGFR1 dimer model that is interacting with heparin, the D2 domain of FGFR1 in the FGF1: FGFR1 complex structure in 1EVT was aligned with D2 from 1FQ9 for both copies of FGFR1. Heparins from the 1FQ9 structure were retained in the model. Both copies of FGF1 in the dimer structure were then replaced with the FGF1 AF3 model of engineered FGF1 with structurally disordered N- and C-termini.

### Cell Fractionation

MCF-7 FGFR1 cells were seeded in 6 well plates by 3 × 10^5^ cells per well and cultured for 2 days. Then, cells were changed to the corresponding serum-free medium and kept for another 24 h. FGF1 translocation inhibition was performed by adding 100 μM bafilomycin, 10 nM radicicol, 10 μM SB203590 inhibitor in the well 20 min before eFGF1-^161^Tb treatment. After incubated with 300 ng/mL eFGF1-^161^Tb in complete media containing 10 U/mL heparin for 6 h, cells were washed by PBS buffer 3 times and lysed with 500 μL nucleus collection buffer (0.1 M NaCl, 10 mM Na_2_HPO_4_, 1% Triton X-100, 1 mM EDTA). Cell nuclei were separated from the cytoplasmic fraction by centrifugation at 20,000 *g* for 2 min and washed once with the same lysis buffer. Radioactivity of each fraction was measured with a HIDEX Gamma counter.

## Supporting Information Available

Structural model of eFGF1; amino acid sequence of eFGF1; coomassie blue gels of eFGF1; fluorescence spectroscopy of eFGF1; analysis of downstream signaling pathways; Western blot of fluorophore-labeled eFGF1; expression levels of FGFR1 and FGFR4 in relevant cell lines; accumulation of eFGF1-^161^Tb in the nuclear fraction of cells; spheroids after treatment with eFGF1-^161^Tb or eFGF1-DOX; and evaluation of eFGF1-DOX internalization (PDF)
