# Supplementary material for: Preparation and Characterization of an Engineered FGF1 Conjugated to 161Tb for Targeting of FGFRs
Source: ACS Omega. 2025 Feb 6;10(6):5730–43. doi: 10.1021/acsomega.4c09179 (PMC11840634; doi:10.1021/acsomega.4c09179)
Supplement: Supplementary file 1 — ao4c09179_si_001.pdf [file ao4c09179_si_001.pdf]

Supporting information

Preparation and characterization of an engineered FGF1 conjugated to ^{161}Tb for targeting of FGFRs

Linlin Song [1, 2], Michal Kostas [1, 2], Jon K. Laerdahl[3,4], Marie Skálová[5], Tereza Janská[5], Asta Juzeniene[6], Svein Ræstad[7], Alexander Krivokapic[7], Georgios N. Kalantzopoulos[7], Jaroslav Soltes[8], Martin Vlk[5], Jan Kozempel[5], Sindre Hassfjell[9], Jørgen Wesche[1, 2, 10 *]

1. Department of Tumor Biology, Institute for Cancer Research, The Norwegian Radium Hospital, Oslo University Hospital, Montebello, 0379 Oslo, Norway
2. Centre for Cancer Cell Reprogramming, Institute of Clinical Medicine, Faculty of Medicine, University of Oslo, Montebello, 0379 Oslo, Norway
3. Department of Microbiology, Oslo University Hospital, Rikshospitalet, 0424 Oslo, Norway.
4. ELIXIR Norway, Department of Informatics, University of Oslo, 0316 Oslo, Norway.
5. Czech Technical University in Prague, Faculty of Nuclear Sciences and Physical Engineering, Břehová 7, 110 00 Prague 1, Czech Republic
6. Department of Radiation Biology, Institute for Cancer Research, The Norwegian Radium Hospital, Montebello, 0379 Oslo, Norway
7. Department of Tracer Technology, Institute of Energy Technology, Instituttveien 18, 2007, Kjeller, Norway
8. Centrum výzkumu Řež s.r.o., Hlavní 130, Řež, 250 68 Husinec, Czech Republic
9. Thor Medical, Karenslyst allé 9C, 0278 Oslo
10. Department of Molecular Medicine, Institute of Basic Medical Sciences, University of Oslo, 0372, Oslo, Norway

*****jorgwe@medisin.uio.no

Corresponding Author: Jørgen Wesche - Department of Tumor Biology, Institute for Cancer Research, The Norwegian Radium Hospital, Oslo University Hospital, Montebello, 0379 Oslo, Norway; email: jorgwe@medisin.uio.no

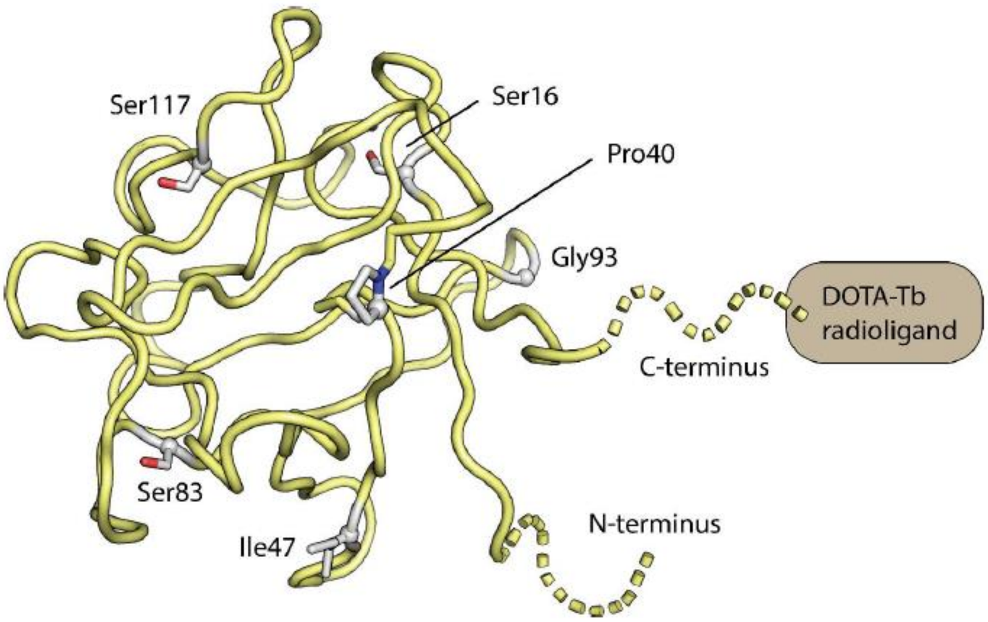

Figure S1. Structural model of engineered C16S Q40P S47I C83S H93G C117S FGF1. The structurally disordered N- and C-termini of the AlphaFold3 model is shown in dashed rendering, while the core segment (residues Lys9 to Ser138) in the model is highly similar to the corresponding part of the experimental FGF1 crystal structure in PDB entry 1EVT (RMSD = 0.49 Å). The location of the DOTA-Tb radioligand at the C-terminal extension is indicated.

MANYKKPKLLYSSNGGHFLRILPDGTVDGTRDRSDPHIQLQLIAESV
GEVYIKSTETGQYLAMDTDGLLYGSQTPNEESLFLERLEENGYNTYIS
KKHAEKNWFVGLKKNGSSKRGPRTHYGQKAILFLPLPVSSDGC

Figure S2. Amino acid sequence of engineered eFGF1 ligand.

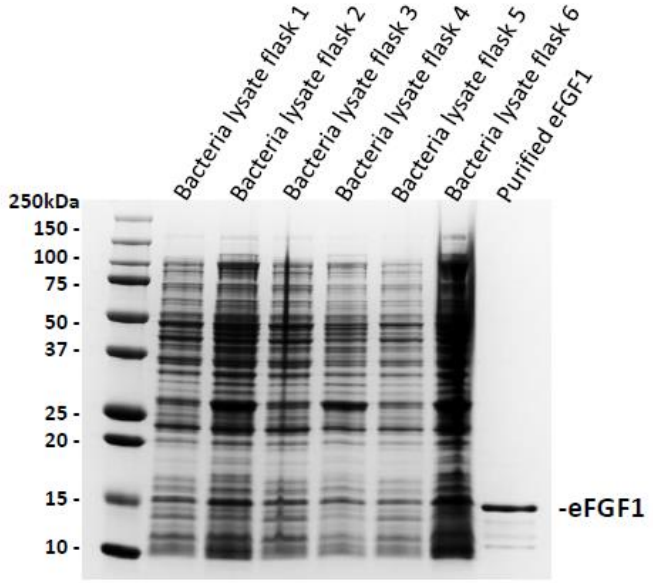

Figure S3. Coomassie blue stained 4-20% gradient PAGE gel of eFGF1 protein expression and purification.

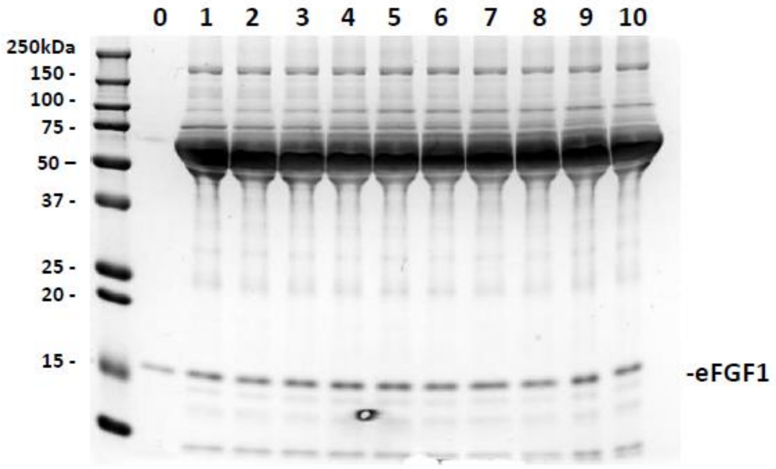

Figure S4. Coomassie blue stained 16% PAGE gel of eFGF1 protein after incubated with complete media at 37°C for 0 to 10 days.

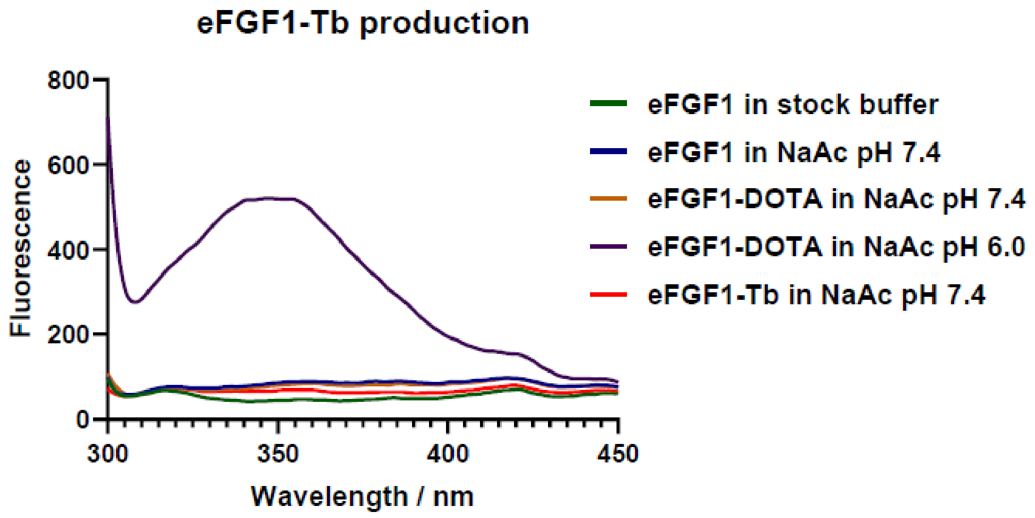

Figure S5. Fluorescence spectroscopy of eFGF1 during each step of conjugation.

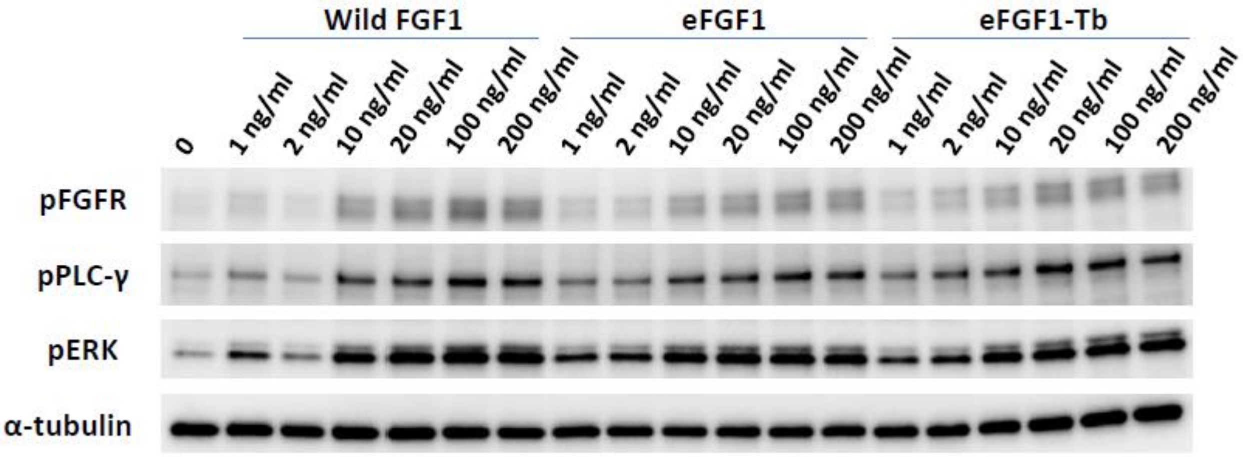

Figure S6. Activation of downstream signaling pathway in MCF-7 FGFR1 cells after stimulation with different concentration of wild type FGF1, eFGF1 and eFGF1-Tb conjugates.

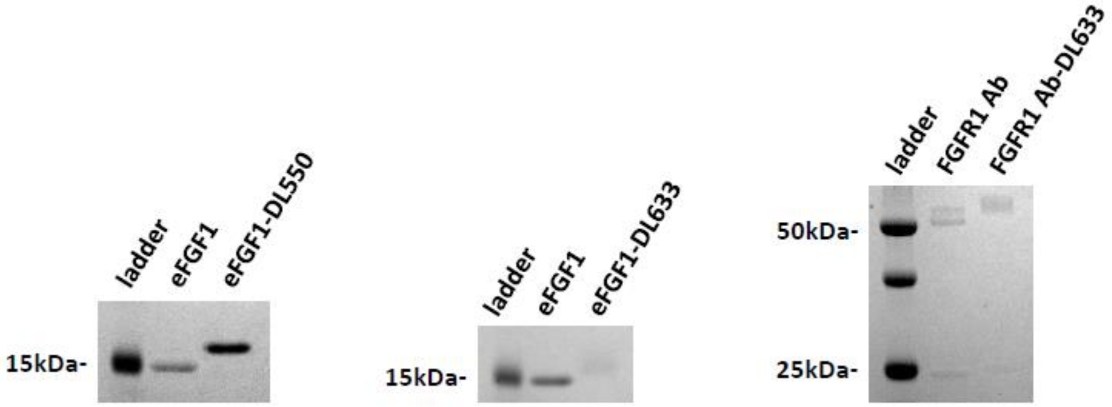

Figure S7. 16% PAGE gel electrophoresis of eFGF1 protein before and after labeling with fluorophore.

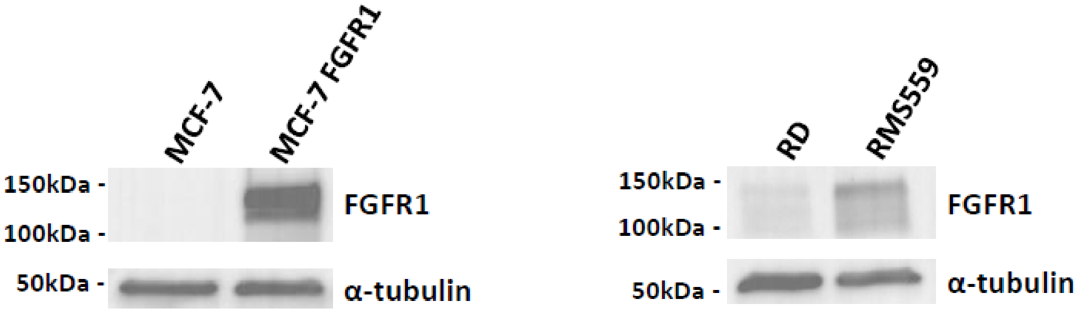

Figure S8. Expression level of FGFR1 in MCF-7, MCF-7 FGFR1, RD and RMS559 cell line. Equal amounts of protein were separated by SDS-PAGE and subjected to Western blotting analysis.

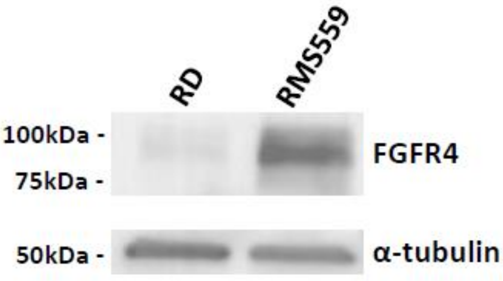

Figure S9. Expression level of FGFR4 in RD and RMS559 cell line. Equal amounts of protein were separated by SDS-PAGE and subjected to Western blotting analysis.

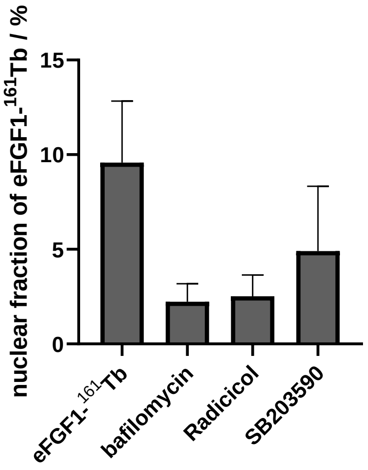

Figure S10. The signal of eFGF1-^{161}Tb accumulated in the nuclear fraction of cells, with or without FGF1 translocation inhibitors (bafilomycin, radicicol, and SB203590). Data are represented as mean values ± SD (from n = 2 independent experimental replicates).

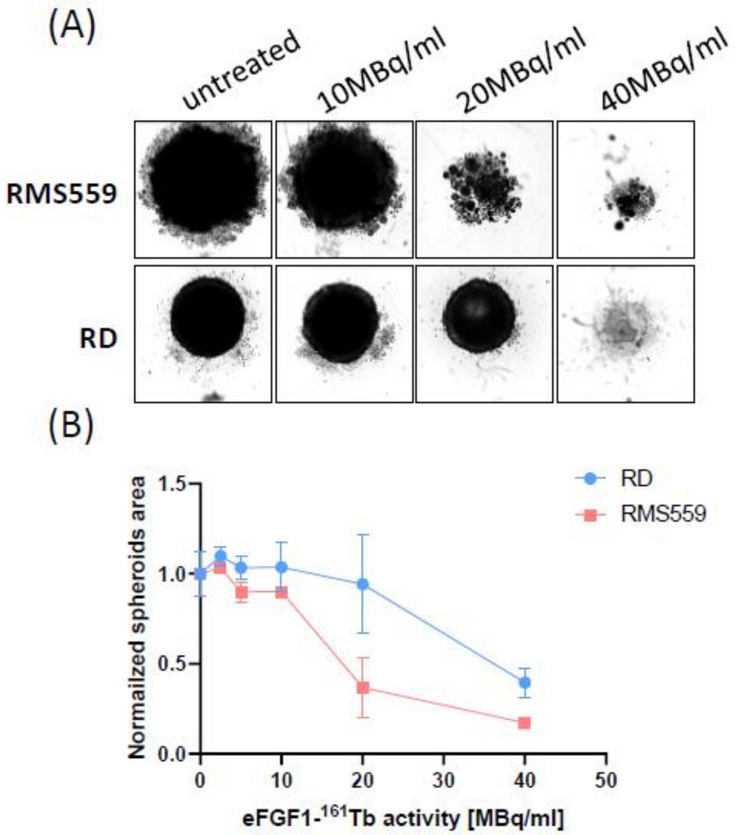

Figure S11. (A) Representative images of RMS559 and RD spheroids after treatment with eFGF1-^{161}Tb radioligands. (B) Spheroids size inhibition by eFGF1-^{161}Tb after treatment with 10, 20 and 40 MBq/ml radioligands for 2 h. Spheroids size was normalized to the untreated group. Data is represented as mean values ± SD (from n = 2 independent experimental replicates).

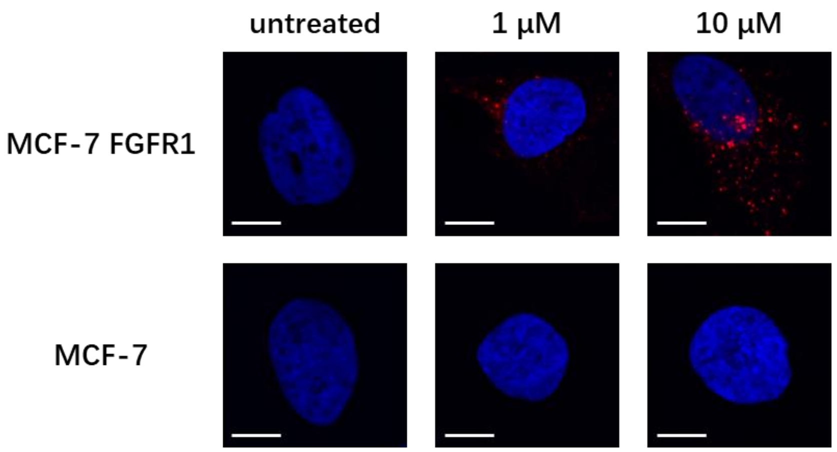

Figure S12. Evaluation of eFGF1-DOX internalization in FGFR overexpressed cells (MCF-7 FGFR1) and MCF-7 cells . (Excitation wavelength: 488 nm for eFGF1-DOX, pseudocolor red; 405 nm for nucleus, pseudocolor blue; scale bars, 10 μm.)

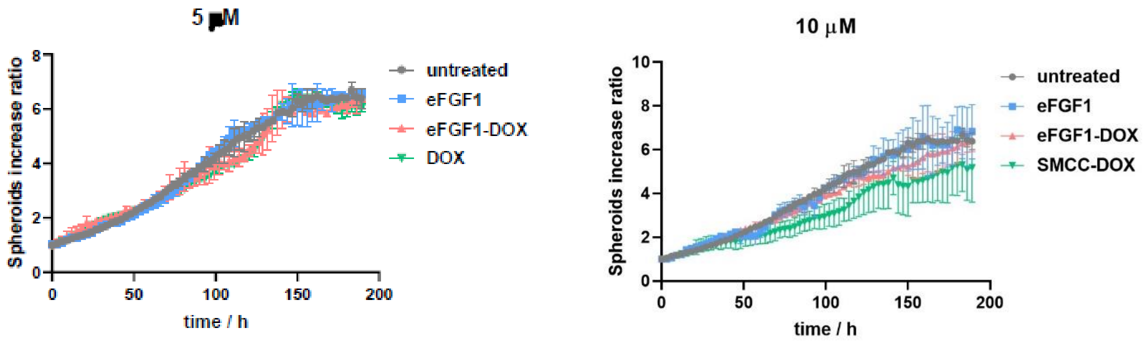

Figure S13. Spheroids size increasing of RMS559 after treatment with 5 and 10 μM eFGF1, DOX-SMCC and eFGF1-DOX. Data is represented as mean values ± SD (from n = 2 independent experimental replicates).
